# A link between agrin signalling and Ca_v_3.2 at the neuromuscular junction in spinal muscular atrophy

**DOI:** 10.1038/s41598-022-23703-x

**Published:** 2022-11-08

**Authors:** Perrine Delers, Delphine Sapaly, Badih Salman, Stephan De Waard, Michel De Waard, Suzie Lefebvre

**Affiliations:** 1 T3S, INSERM, Université Paris Cité, 75006 Paris, France; 2 L’institut du Thorax, INSERM, CNRS, Nantes Université, 44000 Nantes, France

**Keywords:** Neurological disorders, Motor neuron disease, Neuroscience, Diseases, Pathogenesis

## Abstract

SMN protein deficiency causes motoneuron disease spinal muscular atrophy (SMA). SMN-based therapies improve patient motor symptoms to variable degrees. An early hallmark of SMA is the perturbation of the neuromuscular junction (NMJ), a synapse between a motoneuron and muscle cell. NMJ formation depends on acetylcholine receptor (AChR) clustering triggered by agrin and its co-receptors lipoprotein receptor-related protein 4 (LRP4) and transmembrane muscle-specific kinase (MuSK) signalling pathway. We have previously shown that flunarizine improves NMJs in SMA model mice, but the mechanisms remain elusive. We show here that flunarizine promotes AChR clustering in cell-autonomous, dose- and agrin-dependent manners in C2C12 myotubes. This is associated with an increase in protein levels of LRP4, integrin-beta-1 and alpha-dystroglycan, three agrin co-receptors. Furthermore, flunarizine enhances MuSK interaction with integrin-beta-1 and phosphotyrosines. Moreover, the drug acts on the expression and splicing of *Agrn* and *Cacna1h* genes in a muscle-specific manner. We reveal that the *Cacna1h* encoded protein Cav3.2 closely associates in vitro with the agrin co-receptor LRP4*. *In vivo, it is enriched nearby NMJs during neonatal development and the drug increases this immunolabelling in SMA muscles. Thus, flunarizine modulates key players of the NMJ and identifies Ca_v_3.2 as a new protein involved in the NMJ biology.

## Introduction

Spinal muscular atrophy (SMA) is characterized by the degeneration of spinal motoneurons and muscle atrophy. SMA is caused by mutations of the survival motor neuron 1 (SMN1) gene leading to the reduction of SMN protein levels^1,2^. This protein is a ubiquitously expressed DNA/RNA-binding protein involved in the regulation of gene expression, including transcription, splicing, RNA transport and translation, the most characterized function being its role in the U-rich small nuclear ribonucleoproteins (snRNPs) biogenesis^3–5^. In humans, the presence of a copy gene SMN2 provides less fully functional protein due to an alternative splicing of the last coding exon 7^6^. Accordingly, milder SMA phenotypes are observed when the number of copies of SMN2 gene increases^7,8^. The approved SMA therapies are dedicated to increase SMN protein levels using either SMN gene therapy or by targeting SMN2 pre-mRNA with an antisense oligonucleotide (ASO) or a small-molecule. Despite these tremendous scientific and clinical advances, several patients are unable to take those medications, others poorly respond and sometimes, adverse effects are seen^9–13^. Thus, a better understanding of the mechanisms underlying disease should contribute to the emergence of better therapies that are of clinical importance.

Defects of the neuromuscular junction (NMJ) have been observed in the pathogenesis of SMA before motoneuron loss consistent with a “dying-back” phenotype^14–16^. Indeed, delayed maturation, smaller endplate area and endplate denervation are detected in SMA model mice^17–19^. A hallmark of the NMJs is the clustering of muscular acetylcholine receptors (AChRs) on the postsynaptic muscle membrane mediated by the agrin secreted from the motoneurons^20^. Agrin binds the receptor LRP4 and then, the agrin-LRP4 complex binds and activates the transmembrane MuSK promoting AChR clustering through adaptors such as docking protein 7 (Dok7)^21,22^. Moreover, mice lacking functional *Agrn*, *Lrp4* or *Musk* gene die perinatally.

The *Agrn* gene produces multiple isoforms in various tissues using two promoters and alternative splicing of exons encoding domains of the C-terminus, such as the Y and Z sites^23–25^. The alternative first exons encode either the secretory (NtA) or the transmembrane (Tm) N-termini, the latter being mainly expressed in neurons whereas NtA is also expressed in non-neural cells such as muscles^26^. The neural Z^+^ agrin isoforms greatly clusters the AChRs compared to the muscle Z^-^ agrin^23^. As components of the extracellular matrix (ECM), agrins also bind to laminin, heparin, α-dystroglycan and β1 integrins^27,28^. Thus, agrin is also involved in a variety of functions: it maintains the NMJ^29^, promotes heart regeneration^30^, inhibits neurite elongation^31–33^, regulates synaptogenesis in the central nervous system^34^ and coordinates a crosstalk between LRP4/MuSK and integrin-β1 pathways in liver cancer^35,36^.

The role of agrin/LRP4/MuSK/Dok7 signalling pathway in SMA disease was first underlined by the observation that agrin Z^+^ exon is mis-spliced in spinal motoneurons of SMA model mice^37^, that can be corrected by the co-expression of AAV-9 U7-specific Lsm10 and Lsm11 proteins^38^. Modulation of agrin signalling by either the overexpression of a transgenic Z^+^ agrin, injection of a therapeutic agrin or AAV-Dok7 delivery mitigates the phenotype of SMA mice^39–41^. Also, MuSK agonist antibody reduces the neuromuscular defects in SMA mice^42^. Thus, improving the NMJ function through agrin signalling has therapeutic effects on the pathogenesis in SMA model mice. Yet, the expression of the *Agrn* exons other than the Z^+^ exons remains largely unexplored in SMA mice and after treatments.

The implication of NMJs in SMA disease was further illustrated when we reported that flunarizine increases the endplate area and maturation of NMJs and improves disease phenotype in SMA mice, but that independently of the levels of snRNAs and of *Agrn* Z^+^ exons^43^. The exact mode of action on the NMJ remains unknown. Flunarizine was previously used as a T-type calcium channel blocker^44^ and was identified in a chemical screen as a splicing regulator^45^. Both calcium levels and RNA metabolism are altered in SMA models^46–51^. Moreover, ASO treatment of SMA mice mitigates the splicing defects of U12-dependent introns of several genes including *Cacna1h* encoding the T-type calcium channel Ca_v_3.2^47^.

We then envisaged an action of flunarizine on the expression and splicing of *Agrn* and *Cacna1h* genes in skeletal muscles. To address this question, we studied how flunarizine increases the NMJ size in control and SMA model mice. Since the drug effects are muscle-dependent and SMN-independent^43^, we assessed the effects of flunarizine on cultured murine C2C12 myotubes. We report that the drug stimulates in vitro the AChR clustering, an early event in the NMJ formation. This effect has a concentration dependence similar to that of the inhibition of Ca_v_3.2 current suggesting it requires channel block. Moreover, we reveal in vitro and in vivo that the expression and splicing of *Agrn* and *Cacna1h* genes appear to be co-regulated by flunarizine. We also show that the agrin co-receptor LRP4 associates with Ca_v_3.2 that we find localized nearby NMJs in murine neonatal period. Importantly, the Ca_v_3.2 immunolabelling in SMA muscles is increased by flunarizine. Hence, we propose flunarizine effects to associate Ca_v_3.2 with the formation and maturation of perinatal NMJs.

## Results

### Flunarizine enhances the AChR clustering in C2C12 myotubes

**Figure 1:**
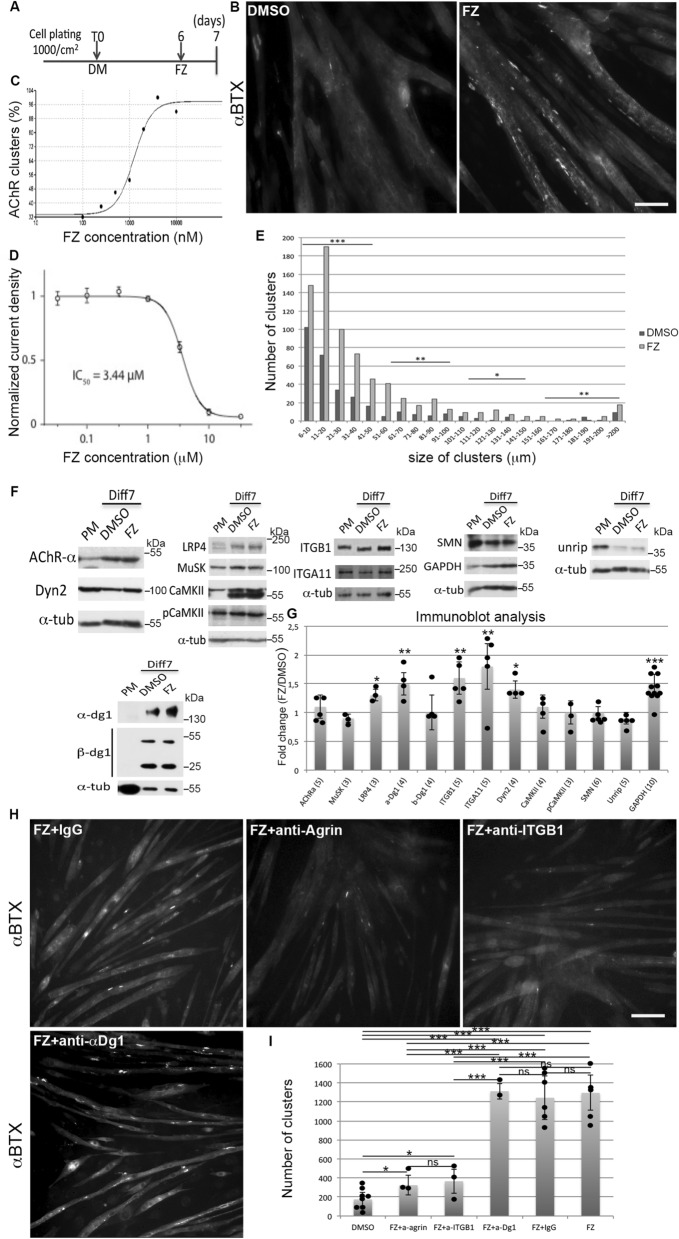
Flunarizine promotes the AChR clustering in the murine C2C12 myotubes. (**A)** Schematic representation of the C2C12 muscle cell cultures and treatment. (**B)** Fluorescence imaging of the AChR clusters with α-bungarotoxin (α-BTX) on DMSO- and flunarizine (FZ)-treated myotubes. (**C)** A flunarizine dose–response curve of AChR clustering (3 ≤ n ≤ 5 for each concentration of 100, 250, 500, 1000, 4000 and 10,000 nM, except 2 for 10 μM). (**D)** Concentration–response curve of the effects of flunarizine on Cav3.2 channels. For each point, mean current density was normalized to mean current density of the vehicle group (7 < n < 24 for each concentration, error bars are SEM). (**E**) Both the size and number of AChR clusters increased with the drug. The columns represent the number of clusters for the same number of myotubes per condition were analysed using ImageJ from 3 independent experiments (345 myotubes per condition). (**F)** Expression of endogenous proteins in total protein extracts of C2C12 myoblasts in proliferation medium (PM) and DMSO- and FZ-treated myotubes in differentiation medium during 7 days (Diff7). Cells were harvested after an overnight DMSO and FZ treatment. The PVDF membrane was immunoblotted with the indicated antibodies with or without a stripping step. α-tubulin serves as a loading control. A 50% increase of GAPDH protein levels is detected relative to the tubulin loading control with the drug. Uncropped images are in supplemental Fig. 3. (**G)** The columns represent fold-changes of protein expression in FZ-treated myotubes compared to the DMSO treatment (1 μl/ml). (**H)** Effects of blocking antibodies to agrin (anti-agrin), integrin β1 (anti-ITGB1) and α-dystroglycan (α-Dg1) on the AChR clustering in the presence of flunarizine (FZ) compared to negative control IgG. (**I)** Columns represent the number of clusters (size > 5 μm) from 40 randomly selected fields of each condition per experiment. Error bars represent SD from the mean values of 3 independent experiments. Diluted DMSO is used in control conditions because flunarizine is insoluble in aqueous solution. Khi-2 test, *, P < 0,05; **, P < 0,01; ***, P < 0,001. Scale bar, 30 μm.

We previously showed that flunarizine enlarges post-synaptic NMJs independently of SMN protein levels in control and SMA model mice^43^. This implies that muscles might respond directly to flunarizine. To test this idea, we assessed whether flunarizine stimulates in vitro the formation of AChR clusters in cultured C2C12 mouse myoblasts fused to form myotubes (Fig. 1A). AChR clusters are visualized using a fluorescent α-bungarotoxin (α-BTX). Flunarizine (4 μM) significantly increased 3- to fourfold the number and size of clusters (Fig. 1B,E). Moreover, flunarizine-induced clustering was dose-dependent (Fig. 1C). The half-maximal concentration (EC50) was 1.3 μM and 4 μM gave maximal effects on clustering. We also assessed flunarizine on the T-type calcium channel Ca_v_3.2 in stably expressing HEK293 cells (Fig. 1D). Electrophysiological recordings revealed that a similar drug concentration-dependence is found for flunarizine on Ca_v_3.2 channel activity (IC_50_ = 3.44 µM). Of note, the time course of flunarizine inhibition illustrates that a maximum inhibition by 10 µM flunarizine is reached after a 5 min incubation period (supplemental Fig. 7). Thus, flunarizine stimulates AChR clustering most probably through a direct action on the Cav3.2 channel, suggesting a direct role of muscle cells in the beneficial effects of the drug in SMA model mice.

### Flunarizine modulates NMJ-related protein levels in C2C12 myotubes

To dissect the flunarizine mechanisms inducing AChR clustering, we examined the protein levels of key NMJ molecules focusing on AChRα subunit, Lrp4, MuSK, dystroglycans (dg), Ca^2+^/calmodulin-dependent protein kinase II (CaMKII), integrin β1 and α11 in C2C12 myotubes (Fig. 1F,G). No effect of flunarizine (4 μM) was detected on the protein levels of AChRα, MuSK and β-dystroglycan whereas 30, 50, 60 and 70% increases were observed for Lrp4, β1 and α11 integrins and α-dystroglycan epitope respectively. We also considered whether dynamin2 (Dyn2) overexpression induces AChR clustering^52^ and regulates NMJ development^53^. An increase of Dyn2 and GAPDH protein levels was shown with flunarizine. No drug effects were observed on CaMKII protein levels or phosphorylation, ruling out a possible involvement of CaMKII, which also regulates the AChR expression and clustering^54,55^. SMN and Unrip (another SMN-complex component) protein levels were unchanged, as expected^56^. Thus, flunarizine may promote agrin signalling since Lrp4/MuSK, β1 integrins and α-dystroglycan are all agrin receptors involved in NMJ biology^25^.

The only source of agrin in our experiments comes from C2C12 myotubes. We then asked whether antibodies that block agrin, integrin-β1 or α-dystroglycan affected the flunarizine-induced AChR clustering. The myotubes were treated with the drug and anti-agrin mouse monoclonal antibody Mab5204^35^ or the anti-integrin-β1 mouse monoclonal antibody Mab1965^57^ or the anti-α-dystroglycan mouse monoclonal antibody IIH6C4^58^ or a negative control mouse antibody (Fig. 1H,I). Under our conditions, Mab5204 and Mab1965 antibodies markedly inhibited AChR clustering, but not IIH6C4 and control antibodies. Besides, α-dystroglycan localized to all AChR clusters in flunarizine-treated myotubes (Supplemental Fig. 4), further supporting a role in cluster stabilization rather than formation^59^. Thus, flunarizine-induced AChR clustering depends on agrin and integrin-β1 in vitro.

### Flunarizine influences Agrn gene expression in C2C12 myotubes and skeletal muscles

**Figure 2:**
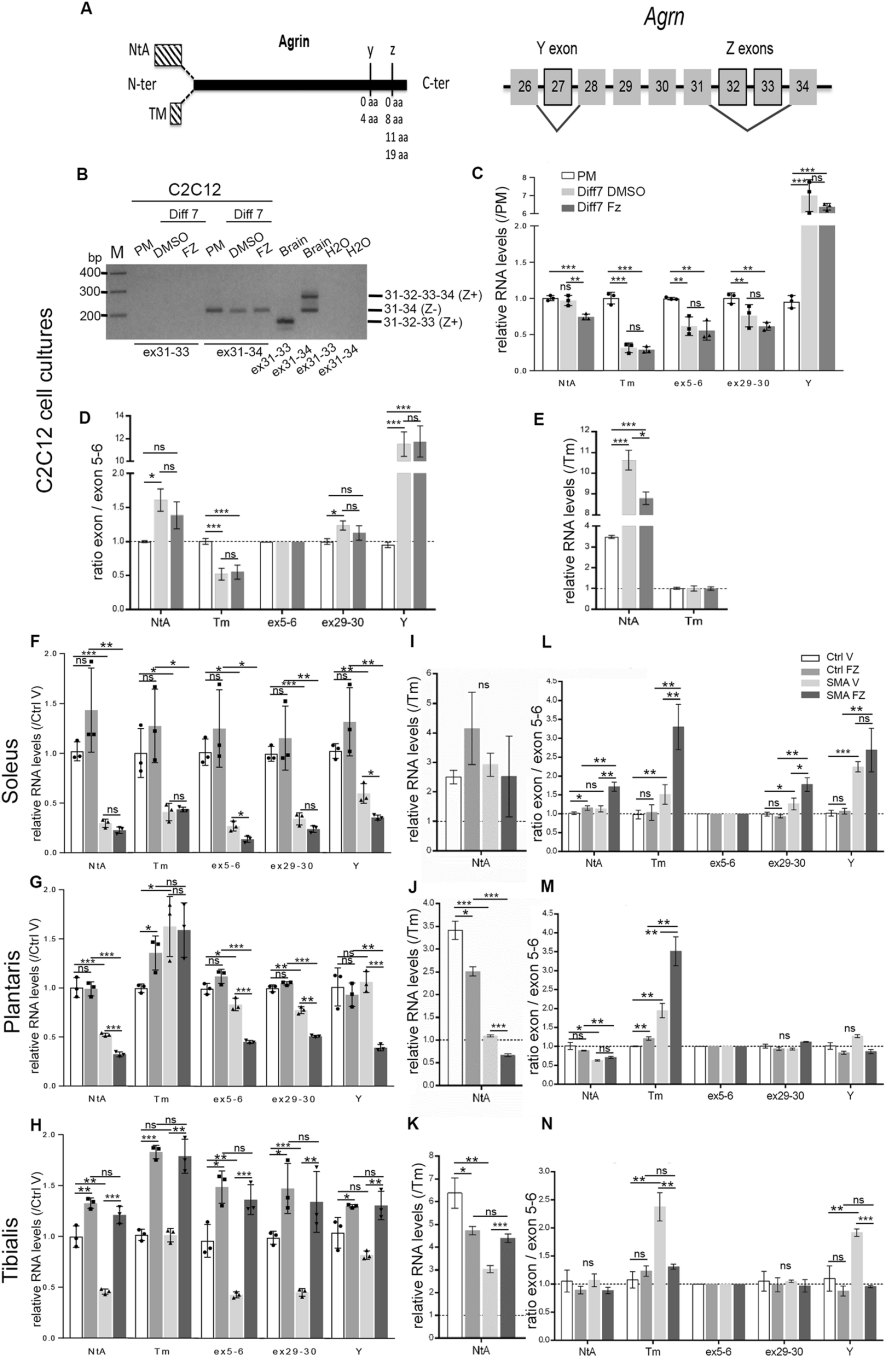
Effects of flunarizine on the expression and splicing profile of *Agrn* gene in C2C12 myotubes and skeletal muscles of SMA model mice. (**A)** Schematic representation of the agrin structure showing the alternative first exon usage of the soluble basal lamina-associated agrin (NtA) or the membrane-spanning domain and a short intracellular region (TM) in the N-terminal end and the splice sites “Y” and “Z” of the C-termini with a schematic representation of the Y and Z exons of the *Agrn* gene. (**B)** Representative analysis by RT-PCR on agarose gel of the *Agrn* Z exons in DMSO- and flunarizine (FZ)-treated C2C12 myotubes (Diff7). (**C–E)** RT-qPCR analysis of the expression of NtA, Tm, ex5-6, ex29-30 and Y exons of *Agrn* gene is performed in C2C12 myotubes compared to myoblasts in proliferation medium (PM). Total RNA is prepared from C2C12 myoblasts grown in proliferating medium (PM) or after 7 days in differentiation medium (D7) with either DMSO or flunarizine (FZ) for the last 16 h. RNA levels in (**C)**, expression ratios between exons and ex5-6 RNA levels in (**D**) and proportion of NtA versus TM in (**E**) are calculated. Ex5-6 is included in all *Agrn* mRNAs. PM was given an arbitrary value of 1 in C and D. (**F–N)** RT-qPCR analysis of the expression of different exons of the *Agrn* gene is performed in the soleus (**F**), plantaris (**G**) and tibialis (**H**) of control and SMA model mice at P10. Control vehicle is given an arbitrary value of 1. Proportions of first exons NtA versus Tm are calculated for the soleus (**I**), plantaris (**J**) and tibialis (**K**). Expression ratios between exons and ex5-6 levels are calculated for the soleus (**L**), plantaris (**M**) and tibialis (**N**). Three genes are used as controls for normalization in C2C12 (*Ppia, Gapdh, 5S or Dusp6, Rragc, 5S*) and skeletal muscles (*Rpl13a, Ppia, Myh4*). Error bars represent SD from the mean values of triplicates from 3 independent experiments with C2C12 cells and 3 mice per group. *NS* not significant (P > 0.05), *P < 0.05, ** < 0.01, *** P < 0.001. Student’s t test.

Results presented so far indicated that flunarizine potentiates the AChR clustering activity of C2C12-derived agrin. We then evaluated whether the drug might impact on muscle *Agrn* gene expression and its spliced isoforms. Agrins are expressed from two different transcriptional start sites giving rise to two N-termini, secreted NtA or transmembrane TM, followed by a common sequence and the alternative Y and Z exons encoding the C-terminal region which contains the binding sites for integrin-β1 and LRP4, respectively^28^ (Fig. 2A). Knowing that the Z-site greatly enhances the AChR clustering^60^, it was possible that flunarizine could promote the inclusion of Z exons. In fact, we illustrate that they are neither present nor induced by the drug in C2C12 myotubes, ruling out this possibility (Fig. 2B). Thus, we found that flunarizine-induced AChR clustering is independent of the Z^+^ agrin.

To uncover whether other *Agrn* isoforms might be produced with flunarizine, we designed RT-qPCR primers for *Agrn* exons, namely NtA, Tm, ex5-6 (included in all isoforms), ex29-30 (3’ region of all isoforms) and the inclusion of the Y-insert (Supplemental Table 2). C2C12 differentiation was associated with a decrease in total *Agrn* mRNA levels (ex5-6) and increased inclusion of the Y-insert (Fig. 2C). The NtA levels were unchanged upon differentiation but Tm levels were markedly reduced. Flunarizine reduced by ≈25% the NtA exon levels in C2C12 myotubes with the exon/ex5-6 ratios being unchanged (Fig. 2D). Furthermore, the proportion of NtA versus Tm showed that there is more NtA than Tm isoforms (as expected) and that an increase of NtA over Tm isoforms is observed upon differentiation as well as a reduction by flunarizine (Fig. 2E). Thus, we found that flunarizine-induced AChR clustering correlates with Y^**+**^Z^**−**^ *Agrn* isoforms in C2C12 myotubes.

To investigate *Agrn* expression in skeletal muscles, we explored *Agrn* exon levels in three hindlimb muscles, namely soleus, plantaris and tibialis from vehicle (V)- and flunarizine (FZ)-treated control (heterozygotes) and SMA model mice at post-natal day 10 (P10). These muscles differ in term of maturation, as indicated by the proportion of embryonic and neonatal myosin isoforms, and by the composition in type I and type II fibers^43^. The maturation of two extensor muscles (soleus and plantaris) is altered in SMA model mice whereas the flexor tibialis is less affected as indicated by the expression of neonatal myosin heavy chain^17,43^. We chose a time point to reflect specific effects rather than ante-mortem changes due to mutant death at ≈12 days^43^. A marked reduction of 60 to 80% in *Agrn* ex5-6 levels was observed in SMA tibialis and soleus respectively, while a modest ≈15% reduction was found in SMA plantaris compared with controls (Fig. 2F–H). Both NtA and Tm exons were reduced in SMA soleus while only NtA was in SMA plantaris and tibialis, indicating a deficiency of the secretory isoforms in SMA muscles restored with flunarizine only in tibialis. Besides, flunarizine similarly increased *Agrn* exon levels in control and SMA tibialis. NtA exon was preferentially expressed over Tm in controls and SMA soleus and tibialis, but equally found in SMA plantaris (Fig. 2I–K). Although ex5-6 levels were reduced in flunarizine-treated SMA soleus and plantaris, the Tm/ex5-6 ratio was increased indicating that the two transcriptional start sites are not similarly sensitive to the drug (Fig. 2L–N). Also, the Y exon/ex5-6 ratio showed with flunarizine a value similar to controls in SMA plantaris and tibialis, but not in soleus. Thus, expression of *Agrn* isoforms is altered in SMA mice in a muscle-specific manner that flunarizine modulates.

### Flunarizine changes Cacna1h gene expression in C2C12 myotubes and skeletal muscles

**Figure 3:**
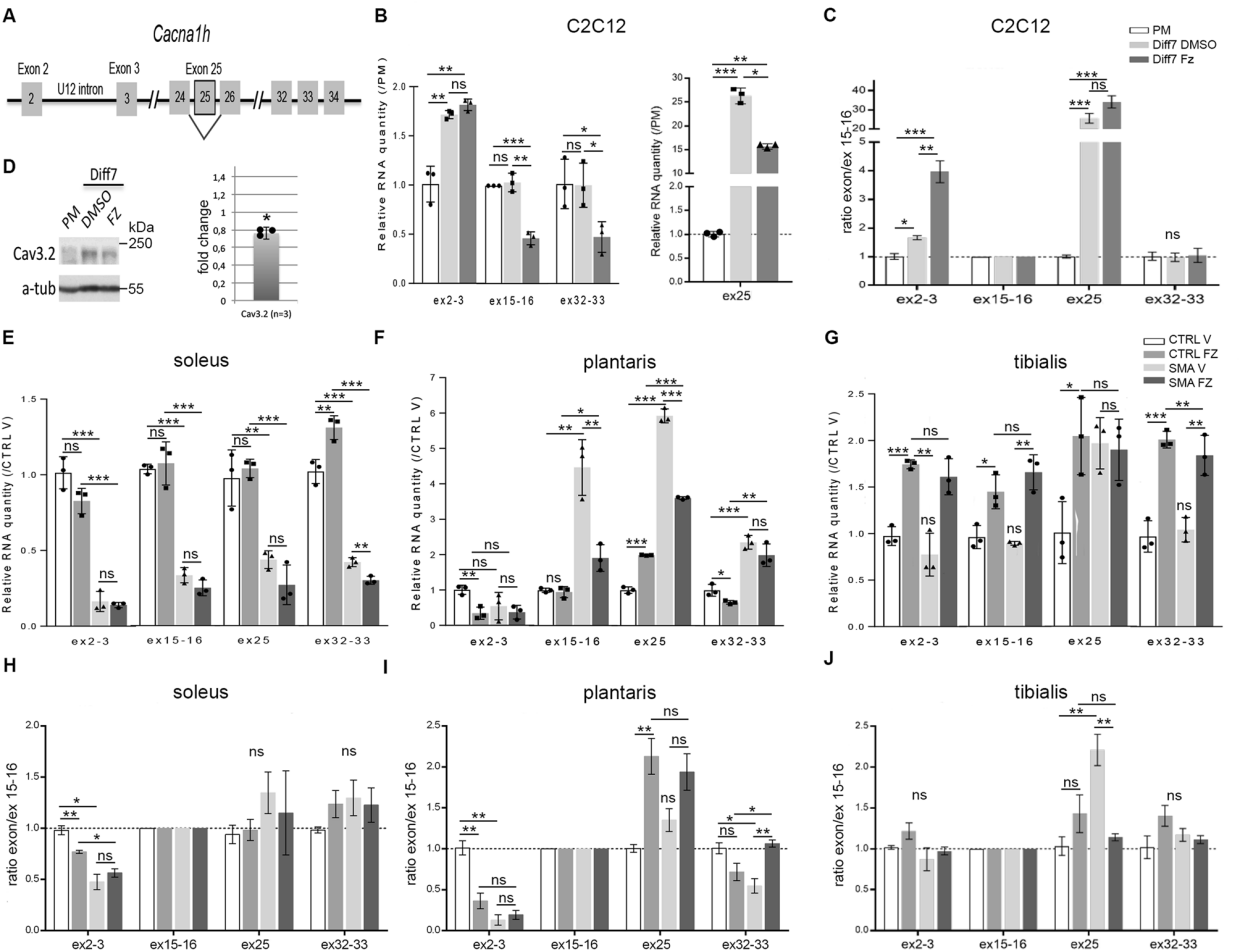
Effects of flunarizine on the expression and splicing profile of *Cacna1h* gene in C2C12 myotubes and skeletal muscles of SMA model mice. (**A)** Schematic representation of the U12-intron flanked by exon 2 and 3 (ex2-3) and the alternative spliced exon 25 of Cacna1h gene is shown. (**B)** RT-qPCR analysis of the RNA levels of ex2-3, ex15-16, ex25 and ex29-30 exons of *Cacan1h* gene is performed in C2C12 myotubes compared to myoblast in proliferation medium (PM). (**C)** The expression ratios between exons and ex15-16 RNA levels, ex15-16 being present in all *Cacna1h* mRNAs. Total RNA is prepared from C2C12 myoblasts grown in proliferating medium (PM) or after 7 days in differentiation medium (Diff7) with either DMSO or flunarizine (FZ) for the last 16 h. (**D)** Immunoblot analysis of the *Cacna1h* encoded protein Cav3.2 confirms a significant 20% reduction of the protein levels in total protein extracts of C2C12 myotubes by flunarizine (n = 3). Uncropped images are found in supplemental Fig. 3. (**E–G**) RNA levels of *Cacan1h* exons are determined at P10 in the soleus, plantaris and tibialis, respectively. (**H–J**) Expression ratios between exons and ex5-6 RNA levels are calculated for the soleus, plantaris and tibialis, respectively. Error bars represent SD from the mean values of triplicates from 3 independent experiments with C2C12 cells and 3 mice per group. NS, not significant (P > 0.05), *P < 0.05, ** < 0.01, ***P < 0.001. Student’s t-test.

The lack of full correlation between the expression levels of *Agrn* isoforms (Fig. 2) and flunarizine action on NMJ size^43^ suggest that additional events contribute to the drug effects. Because flunarizine is classified as a blocker of T-type calcium channels Ca_v_3.1, Ca_v_3.2 and Ca_v_3.3 encoded by *Cacna1g*, *Cacna1h* and *Cacna1i* genes, respectively^44^, we then investigated whether the drug could modulate *Cacna1h* expression. Of note, *Cacna1g* and *Cacna1i* transcripts were below our revelation limits and are thus not detectable. Thus, primers were designed and validated for the analysis of expression and splicing of *Cacna1h* gene: ex2-3 (exons flanking an U12-dependent intron), ex15-16 (included in all *Cacna1h* mRNAs), ex25 (alternative spliced exon 25) and ex32-33 (3’ region in all *Cacna1h* mRNAs) primers (Supplemental Table 2, Fig. 3A). Ex15-16 levels were similar between C2C12 myoblasts and myotubes whereas ex2-3 and ex25 levels increased with differentiation (Fig. 3B). Flunarizine reduced ex15-16 and ex25 levels by 50 and 40% respectively, but had no effect on ex2-3 levels in myotubes. We further evaluated exon/ex15-16 ratios (Fig. 3C). The expected value of 1 was obtained with the drug for ex32-33/ex15-16 ratio whereas a ≈ 3-fold increase was found for ex2-3/ex15-16 ratio, indicating a relative increased splicing of U12 intron in *Cacna1h* transcripts. Moreover, we showed a 25% reduction of Ca_v_3.2 protein levels in flunarizine-treated C2C12 myotubes (Fig. 3D). Our results also confirmed the specificity of the antibodies used herein. Because U12 introns are removed more slowly than U2 introns, they dictate the expression levels of mature transcripts^61^. Thus, flunarizine decreases in vitro* Cacna1h* mRNA levels that are partially compensated by U12 intron removal. Minor snRNA gene delivery improves disease phenotype in SMA mice^62^. Although snRNA levels were unchanged with the drug (Supplemental Fig. 1), our data associate minor U12 spliceosomes as potential targets of the drug in C2C12 myotubes.

Next, we measured *Cacna1h* gene expression in skeletal muscles of control and SMA mice (Fig. 3E–J). We showed that ex15-16 levels in SMA mutants as well as the effects of flunarizine on controls and mutants were muscle-specific. Indeed, a threefold reduction of ex15-16 levels was observed in SMA soleus while a ≈ 5-fold increase was found in SMA plantaris when compared to controls (Fig. 3E–G). Flunarizine reduced the elevated ex15-16 levels in SMA plantaris but did not restore levels in SMA soleus. Likewise, to what was observed with the *Agrn* gene (Fig. 2H), SMA tibialis expressed as much *Cacna1h* mRNA levels as controls and increased similarly with the drug (Fig. 3G). Alternative ex25 levels were increased in SMA plantaris and tibialis compared to controls whereas they were reduced in SMA soleus. Flunarizine corrected the relative ex25 inclusion in mRNAs of SMA tibialis but increased it in control plantaris. The ratio ex2-3/ex15-16 remained low in flunarizine-treated SMA soleus and plantaris, indicating that the retention of the U12 intron persisted. Importantly, ex32-33/ex15-16 ratio showed a value of 1 in flunarizine-treated SMA plantaris, suggesting an increase of full-length mRNAs with the drug.

### Ca_v_3.2 protein is enriched nearby NMJs and with flunarizine in SMA model mice

**Figure 4:**
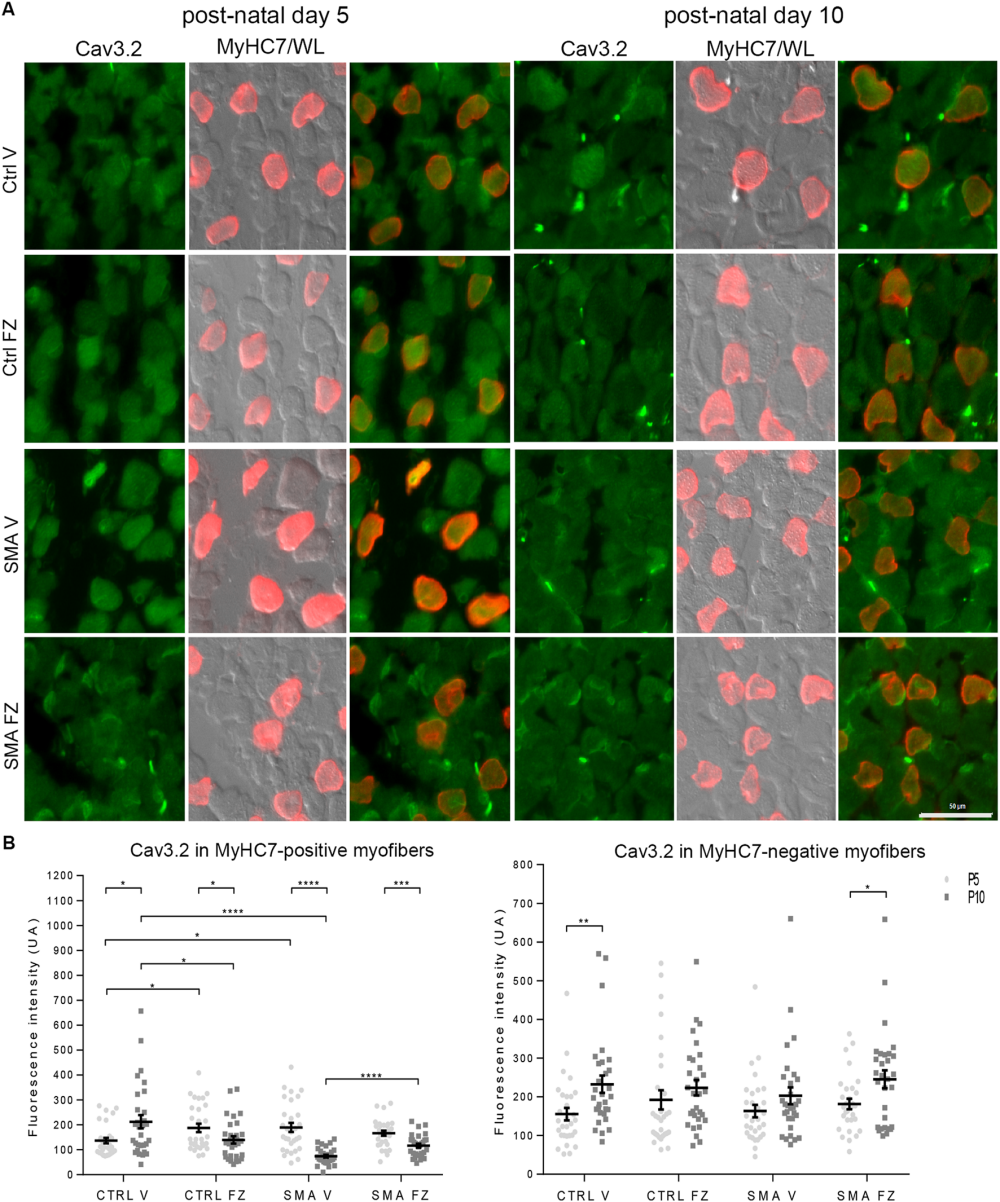
Flunarizine prevents the reduction of myofiber Cav3.2 immunolabelling during neonatal development in SMA mice. (**A)** Muscle fiber immunostaining with Cav3.2 (green) and MyHC7 (red) is shown for the plantaris from vehicle (V)- and flunarizine (FZ)-treated control and SMA mice at P5 and P10, respectively. The original images are presented in supplemental Fig. 8. (**B)** Normalized fluorescence intensity of individual muscle fibers for Cav3.2 detected in panel A is shown. Each symbol represents total value of fluorescence intensity in a fiber. The mean value of fluorescence is in supplemental Fig. 9. The distribution for the 4 groups of mice of MyHC7 immunolabelled or unlabeled fibers is compared using Student’s t-test. *NS* not significant (P > 0.05), *P < 0.05, **P < 0.01, ***P < 0.001 (3 mice per group). Scale bar, 50 μm.

Ca_V_3.2 current has been detected in murine neonatal myofibers^63^. We then asked whether the distribution of Ca_v_3.2 in myofibers was impacted by flunarizine. To this end, we performed co-labelling immunofluorescence experiments using anti-type I MyHC7 (slow Type I fibers) and anti-Ca_v_3.2 antibodies on serial sections of control and SMA plantaris at P5 and P10 (Fig. 4A, Supplemental Fig. 8). The SMA mice start developing symptoms at P5^43^. The anti-Ca_v_3.2 antibodies revealed a cytoplasmic and membranous labelling of myofibers. Reduction of total fluorescence in Type I myofibers between P5 and P10 was observed in all conditions, except for the vehicle-treated controls (Fig. 4B). The flunarizine-treated SMA type I fibers recovered cytoplasmic fluorescence at P10 compared to vehicle-treated SMA fibers. Flunarizine had no effects on total Ca_v_3.2 fluorescence intensity of MyHC7-negative fibers (presumably rapid type II) in controls whereas an increase was observed in those SMA fibers. Thus, each fiber type responds differently to flunarizine.

**Figure 5:**
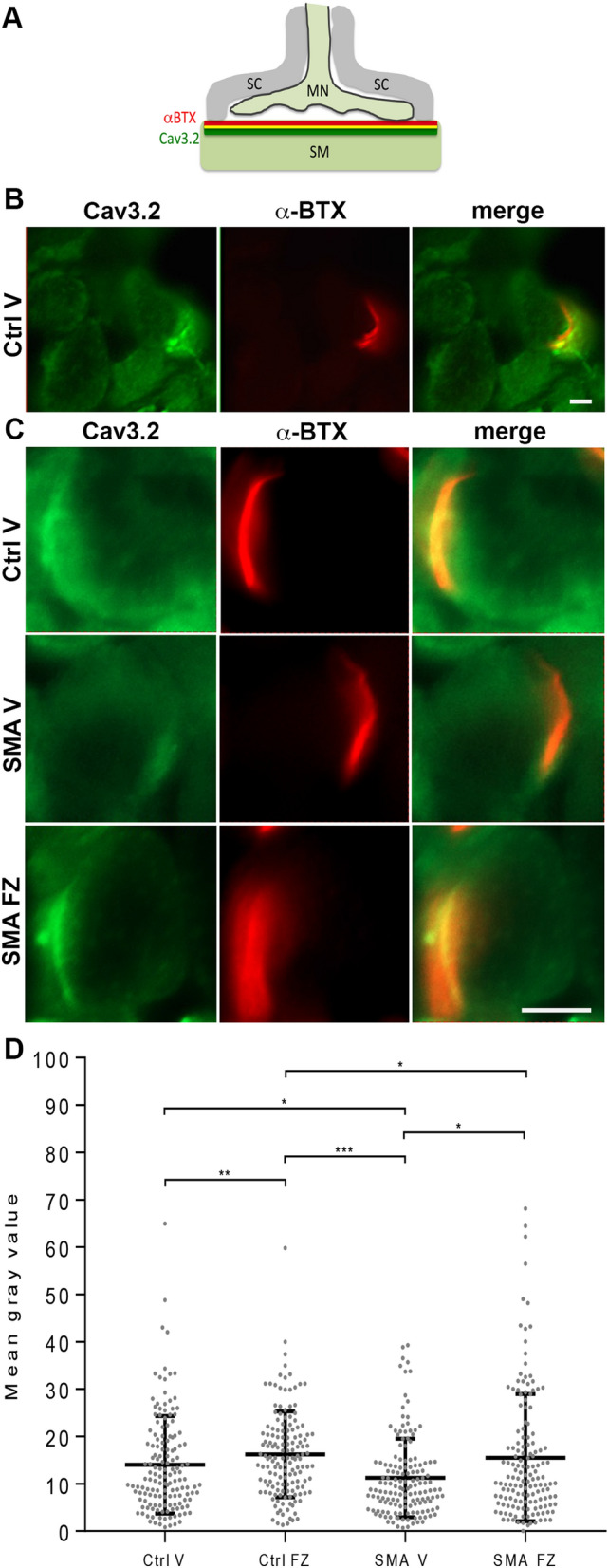
The Cav3.2 protein is enriched nearby the neuromuscular junction and its deficiency is improved by flunarizine in muscles of SMA mice. (**A)** Schematic representation of the Cav3.2 immunolabellling at post-synaptic NMJ. *MN* motor neuron, *SC* Schwann cells, *SM* skeletal muscle. (**B)** Fluorescence experiment of Cav3.2 reveals labelling close to postsynaptic sites of a NMJ using the α-BTX staining of AChRs and within its motor neuron in cryostat sections of P5 plantaris from vehicle-treated control mice. The image is focused α-BTX staining. (**C)** Fluorescence experiment uncovers the accumulation of Ca_v_3.2 nearby postsynaptic sites of the NMJs as evidenced with the α-BTX staining of AChRs in cryostat sections of P10 plantaris from control mice (upper panel), the reduction (middle panel) and restoration in SMA mice with flunarizine (bottom panel). Scale bar, 10 μm. (**D)** Normalized mean fluorescence intensity of individual NMJ for Ca_v_3.2 detected in panel B is shown. Each symbol represents the mean value of fluorescence intensity of indivdual NMJ. The fluorescence of the vehicle-treated control fibers at P5 is given an arbitrary value of 1. The distribution for the 4 groups of mice of Ca_v_3.2 immunolabelled NMJ is compared using the Mann–Whitney test using GraphPad. NS, not significant (P > 0.05), *P < 0.05, **P < 0.01, ***P < 0.001 (3 mice per group, total of 600 NMJs).

Previous studies have shown Ca_v_3.2 expression in motor neurones^64,65^, blood vessels^66^, muscle sensory afferents but not in Schwann cells^67^, and all these cell types being present in muscle tissues. We focused here on the Ca_v_3.2 membranous labelling of myofibers and NMJs looking at Ca_v_3.2 labelled muscle sections at P10 counterstained with a fluorescent α-BTX to label AChRs (Fig. 5A–D). Our immunofluorescence experiments showed in P5 and P10 muscle sections that the immunoreactivity of Ca_v_3.2 was close to that of the labelling with the fluorescent α-BTX, indicating that Ca_v_3.2 is present nearby NMJs. We also showed that flunarizine enhanced Ca_v_3.2 immunostaining nearby NMJs in SMA muscles (Fig. 5D). Thus, defective Ca_v_3.2 distribution in myofibers might contribute to SMA pathogenesis.

### Flunarizine modulates protein interactions in C2C12 myotubes

**Figure 6:**
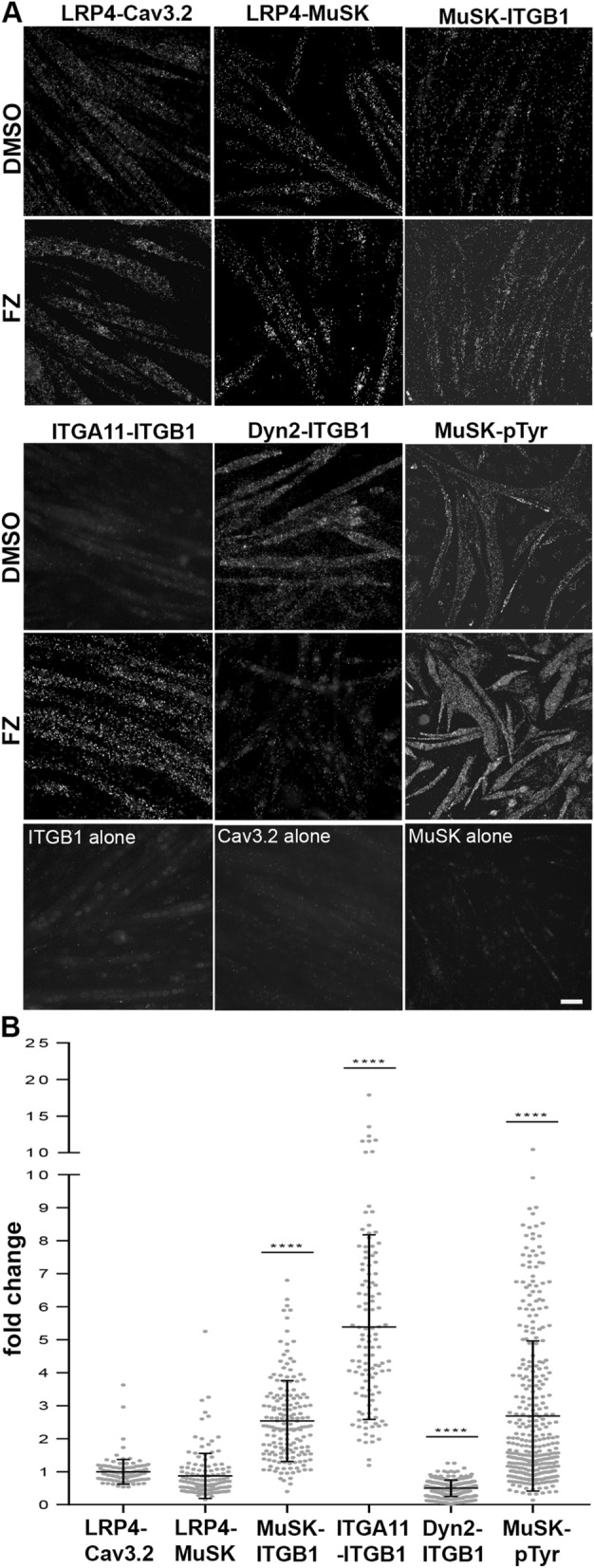
Visualization of key NMJ protein interactions in flunarizine-treated C2C12 myotubes. (**A)** In situ PLA detection of endogenous protein interactions in C2C12 myotubes with specific antibodies for the following protein pairs: LRP4-MuSK, LRP4-Cav3.2, MuSK-integrin β1, integrin α11-β1, Dyn2-integrin β1 and MuSK-phosphotyrosines. As negative controls, one primary antibody is omitted or replaced by a negative control antibody (DAKO). The PLA positive signals are shown as bright dots. (**B**) Fold change in mean fluorescence intensity of individual C2C12 myotubes detected in panel A is shown. The number of myotubes analysed with ImageJ is found in supplemental Table 3. Scale bar, 30 μm.

To examine whether flunarizine modulates protein interaction between Ca_v_3.2 and the agrin-LRP4-MuSK pathway, the C2C12 myotubes were treated with the drug, fixed and evaluated using in-cell co-immunoprecipitation protein ligation assay (PLA) with antibodies generated in different species and specific for each protein (Fig. 6). Focusing on proteins critical for the AChR clustering, we tested the following pairs: LRP4-MuSK, LRP4-Ca_v_3.2, MuSK-integrin β1, MuSK-phosphotyrosines, integrins α11-β1 and Dyn2-integrin β1. The PLA positive signals between two proteins in close proximity (< 40 nm) appeared as fluorescent dots that can be compared between DMSO- and flunarizine-treated C2C12 myotubes. Specific PLA signals were detected for all combinations. The positive signals with LRP4-Ca_v_3.2 and LRP4-MuSK combinations were indicative of constitutive protein associations in C2C12 myotubes. We also found that flunarizine enhances the association of integrin-β1 with both MuSK and integrin-α11 whereas Dyn2-integrin-β1 association was reduced (Fig. 6B). Although MuSK phosphorylation was not detected in soluble immunoprecipitation protein fractions (Supplemental Fig. 10), PLA signals generated with MuSK and phosphotyrosines were markedly increased by flunarizine. Thus, flunarizine changes the close association(s) of key NMJ proteins.

### Flunarizine reduces Txnip expression in skeletal muscles of SMA model mice

**Figure 7:**
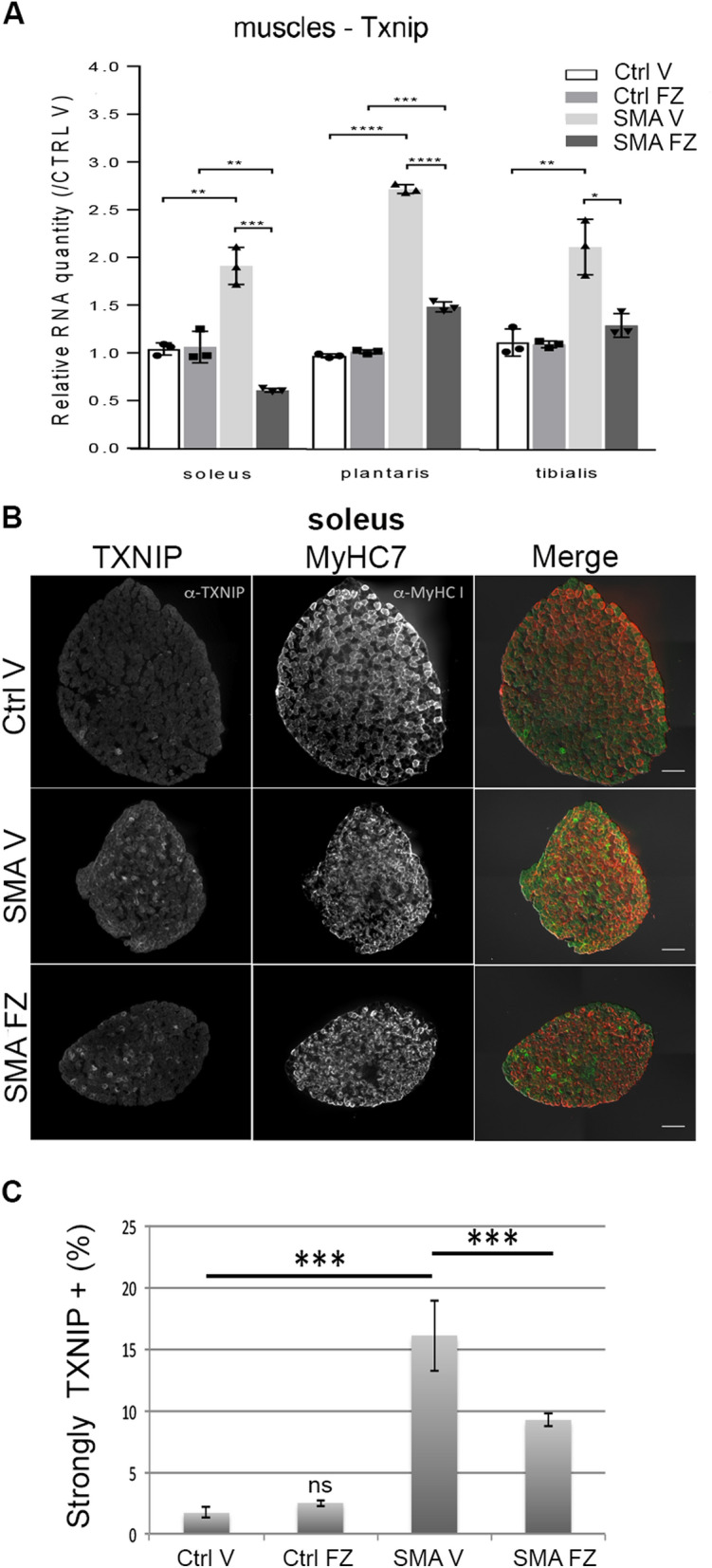
Flunarizine reduces the increased *Txnip* expression levels in skeletal muscles of SMA model mice. (**A)** *Txnip* RNA levels in the soleus, plantaris and tibialis in vehicle- (V) and flunarizine (FZ)-treated controls (Ctrl) and SMA model mice. Vehicle-treated control (Ctrl V) is given an arbitrary value of 1. *NS* not significant (P > 0.05), *P < 0.05, ** < 0.01, ***P < 0.001, Student’s t test (3 mice per group). (**B)** Muscle fiber immunostaining with anti-TXNIP (green) and anti-MyHC7 (red) antibodies is shown for the soleus from vehicle- and flunarizine-treated control and SMA mice at P11. (**C**) Columns represent the percentage of fibers strongly TXNIP-positive (3 mice per group). Error bars represent SD from the mean values. Scale bar, 30 μm.

Oxidative damages might also contribute to NMJ alterations in motoneuron diseases^68^. Flunarizine reduces the pro-oxydant TXNIP in SMA patient fibroblasts^56^ like other calcium channel blockers do in pancreatic beta cells^69^. Interestingly, *Txnip* transcripts were upregulated in muscles with no satellite cells (SC) compared with SC replete muscles^70^ that is reminiscent of reduced SC number in muscles of SMA mice^71–73^. We examined *Txnip* expression in muscles of flunarizine-treated SMA mice (Fig. 7). Flunarizine reduced upregulated *Txnip* mRNA levels in SMA muscles (Fig. 7A). Immunofluorescence co-labelling experiments with antibodies against TXNIP and MyHC7 (type I fibers) revealed that all neonatal myofibers were TXNIP-positive with 2% being strongly labelled in controls while 16% were in the SMA soleus and reduced to 10% with flunarizine (Fig. 7B,C). This fits with high TXNIP expression in disused adult mouse and rat muscles^74^. Thus, flunarizine reduces the elevated *Txnip* mRNA levels in muscles of SMA mice.

## Discussion

Three SMN-restoring therapies exist for SMA patients that improve survival and motor function, but yet patient response to treatments is variable. So, additional therapeutic targets should be identified to optimize existing SMA therapies or implement new ones. We have previously shown flunarizine to increase myofiber size, NMJ area and innervation in SMA model mice^43^. We focused herein on skeletal muscles to assess flunarizine on NMJs. We show that flunarizine can modulate agrin-signalling pathways, involved in NMJ formation and maturation, providing further insight into SMA pathogenesis.

Motoneuron disease mouse models and patients present NMJ defects^75,76^. Synaptic dysfunction is an early event in SMA that generates changes in sensory-motor circuits^77–79^. SMA is a retrograde neuropathology in which motoneuron degeneration begins at presynaptic NMJs and progresses to cell bodies^15^. In severe SMA mouse models, motoneuron numbers are normal at birth and decrease around post-natal day 3 to 5^80,81^. Post-natal NMJ maturation occurs during the first 3–4 weeks after birth. During this period, NMJs enlarge and their morphology changes from oval to perforated plaques to reach a pretzel-like shape^82^. Ca_v_3.2 current is high in perinatal myofibers, progressively decreases during the 3–4 weeks after birth and is undetectable in adult muscles, except during muscle regeneration and repair^63^. This developmental period has high demands for SMN and is when the motor phenotype caused by SMN deficiency can be rescued by SMN-based therapies^83–85^. It is also when SMN-independent therapies improve disease phenotype^12,43,86–88^.

Stimulated agrin/LRP4/MuSK pathway ameliorates SMA and amyotrophic lateral sclerosis (ALS)^89^. Agrin is a key molecule in post-synaptic NMJ biology. Changes in agrin isoforms impact on NMJ formation, maturation and stability^23^. *Agrn* gene has alternative spliced Y and Z exons. Isoforms with Z-sites (Z^+^) are synaptogenic at NMJs whereas isoforms without them (Z^-^) have little AChR clustering activity *in vivo*^90^. Motoneurons express Z^+^ agrin while muscle cells do not. Z^-^ agrin has also functions: it can modulate AChR phosphorylation^91^, stimulate AChR gene expression^92,93^ and in certain conditions, induce AChR clustering^60,94,95^. Z^-^ agrin can bind myotube cell surface proteins, such as neural cell adhesion molecule, α-dystroglycan, laminins and integrins^28,96–99^. Z^+^ agrin can also bind these proteins, but the α-dystroglycan binds more strongly Z^-^ agrin than Z^+^ agrin. Moreover, the Y-site changes the interactions, the Y^**–**^Z^**−**^ agrin exhibiting the highest binding to α-dystroglycan^90^. The flunarizine-modulated Y-site also implicates α-dystroglycan activity to the response in SMA muscles.

At post-natal day 10, each skeletal muscle is very different in term of maturation. The embryonic MyHC persists for a time after birth. The soleus is the less mature muscle, as a larger proportion of embryonic fibers (60%) than in plantaris (30%) and tibialis (20%) is found^43^. Two *Agrn* promoters produce transcripts with either NtA or Tm first exon encoding secreted or transmembrane isoforms, respectively. Both motoneurons and myofibers express NtA exon, encoding a laminin-binding domain^27^. It has been shown that *Smn*-deficient motoneurons derived from SMA mice do not respond to laminins^100^, which is consistent with a reduction in NtA-agrin. The marked NtA reduction we observed also in SMA muscles indicates altered expression in both cell types. NtA and Tm exons are not equally affected in SMA muscles and respond differently to the drug in a development/muscle-specific manner, the more mature SMA tibialis behaving like controls. Tm exon in vivo is expressed almost exclusively by neurones^27^ and in muscles, by neurones innervating myofibers. Different Tm exon levels are observed among SMA muscles, suggesting a difference in skeletal motoneurons^101^ and/or in retrograde signals from the myofibers^14^.

Although a threshold Tm exon level correlates with flunarizine effects on NMJ size in SMA plantaris and tibialis^43^, additional mechanisms have to be considered to explain how it occurs in SMA plantaris (similar Tm levels between vehicle and flunarizine). It has been shown that the Y-site modulates Tm-agrin at excitatory synapse-like specializations in the central nervous system^34^. These authors proposed that an indirect interaction of Tm-agrin and LRP4 is required to cluster proteins at post-synaptic membrane. It is possible that Ca_v_3.2 could mediate indirect interactions. Other studies have shown that integrin-β1 could mediate the interaction between agrin and α-dystroglycan^28,57^. It is therefore tempting to speculate that Ca_v_3.2 might be an agrin co-receptor in early post-synaptic NMJ differentiation.

Regarding *Cacna1h* gene (encoding Ca_v_3.2), its U12-dependent intron splicing of *Cacna1h* gene is altered in SMA mice^47^. Motoneuron disease ALS and a severe congenital amyotrophy are associated with genetic *Cacna1h* variants as risk factors^102–105^. Interestingly, other types of calcium channels, namely P/Q-type, limit the localization and activation of MuSK and prevent AChR clustering in extra-synaptic domains of myofibers^106^. It is interesting that a blocker of T-type calcium channels can modulate the expression of *Cacna1h* at the RNA and protein levels. The reason might be the low selectivity of the drug for Ca_v_3 channels^107^. It is tempting to speculate that inhibition of T-type channels partially explains the flunarizine effects. The prolonged action of flunarizine on Cav3.2 current inhibition provides support for a role of this channel (Supplemental Fig. 7). Flunarizine might promote a feedback loop on *Cacna1h* expression by altering intracellular Ca^2+^ levels that counteracts NMJ defects in SMA plantaris and tibialis. This is in agreement with the role of altered RNA metabolism in SMA and ALS^108^ and the effects of flunarizine on RNA splicing^45^. Our results have also broad relevance in other human diseases suggesting that flunarizine could be deleterious in young patients affected with a channelopathy caused by dominant-negative Ca_v_3.2 mutations^109^.

Calcium is a key regulator in skeletal muscle biology. AChR cluster formation and maintenance involved intracellular and extracellular calcium^110,111^. In murine muscles, Ca_v_3.2 current peaks around embryonic day E16 that coincide with the late embryonic NMJ maturation step. Indeed, aneural AChR clusters are present at E11-12.5, then innervation takes place (E12.5-E14) and aneural clusters disperse at E14-E17 and only innervated clusters remain on myofibers^20^. Myogenesis also occurs in this period. From E14.5 to birth, myoblasts adhere and fuse to primary fibers to form secondary myofibers^112^. Ca_v_3.2 current is activated in vitro when myoblasts fuse to form myotubes^113^. This may explain the reduced muscle size in SMA mice. Reduced number of myofibers in SMA soleus and plantaris^43^ correlates here with the reduced *Cacna1h* RNA levels. Flunarizine increases fiber size and number to reduce atrophy of SMA soleus, but only fiber size to correct atrophy in SMA plantaris^43^. Post-natal growth of muscle normally happens by increases in fiber area but not in fiber number^20,114^. These observations might point to a difference in myofiber types and their respective expression of Ca_v_3.2, as seen herein. The soleus presents a 50%–50% proportion of slow type I and fast type II fibers. The plantaris and tibialis contain a small proportion of type I fibers (10–20%) that disappears almost completely during postnatal development. Resting calcium levels are higher in slow than fast fibers. Therefore, calcium transients have different effects in either fibers due to the level differences in proteins controlling calcium homeostasis. Calcium can activate calmodulin-regulated phosphatase calcineurin and can activate calcium-dependent proteases. In slow myofibers, calcineurin regulates the nuclear translocation of transcription factor NFATc1 controlling the slow gene program. Furthermore, increased calcium influx through Ca_v_3.2 induces cardiac calcineurin/NFAT hypertrophic signalling^115^. Given that calcium handling is altered in SMA^50^, flunarizine might modulate the expression of other muscle genes still to be identified in the future.

Our observation of Ca_v_3.2 is found in close proximity to LRP4 in C2C12 myotubes raises the question of its potential localization nearby the NMJs during perinatal development. We can speculate but do not yet fully understand why Ca_v_3.2 is localized close to NMJs. An interesting feature of Ca_v_3.2 is the existence of a PDZ-binding domain in its cytoplasmic C-terminus^116^. PDZ proteins might control calcium channels activity, regulate their plasma membrane density and influence downstream signalling pathways^117^. Several PDZ scaffolding proteins are found at the post-synaptic NMJ, including the neuronal nitric oxide synthetase (nNOS) and α-syntrophin^118,119^, MAGI-1c^120^ and PDZ domain containing RING finger 3 (PDZRN3)^121^. Thus, the NMJ localization of Ca_v_3.2 may be explained by the previous report that the PDZ domain of nNOS interacts in vitro with the PDZ-binding domain of Ca_v_3.2^122^. This is particularly interesting in the context of SMA and ALS, where the immunostaining for nNOS is reduced/abolished at the sarcolemma of atrophic fibers in patient muscle biopsies and preserved at hypertrophic fibers (indicative of innervation) in SMA biopsies^123,124^. This result is noteworthy in many respects with flunarizine effects on SMA mouse muscles. nNOS activity regulates calcium homeostasis^125^, promotes AChR clustering in vivo and enlarges NMJs^126^. Regarding NMJ development, Wnt induction of the AChR clustering is complex. RNA analysis identified Wnt4 and Wnt9a expression in C2C12 myotubes^127^ that are not changed by flunarizine nor do AChR subunits and downstream YAP targets (Supplemental Figs. 5 and 6). Exploring the role of Wnts in flunarizine-induced AChR clustering could provide additional insights in the future. Considering the few dysregulated pathways in skeletal muscles from SMA mouse embryos^128^ with dysregulated muscle pathways after birth^37,88,129,130^, our findings point to the importance in investigating the drug effects during neonatal development in SMA mice. With a better understanding of the responses to flunarizine, it may be possible to extend the existing treatments to combined therapies against motoneuron diseases.

## Materials and methods

### Cell culture, myotube treatment and immunodetection

C2C12 muscle cells were grown on TPP culture dishes in DMEM-glutamax supplemented with 20% fetal calf serum (FCS) and penicillin and streptomycin (100 units/ml) and differentiated in DMEM-glutamax containing 2% horse serum. After 6 days in differentiation medium, myotubes were treated overnight with flunarizine (4 μM, as recommended by Sigma-Aldrich for Lopac library L6912, Cav3.2 calcium current IC_50_) or DMSO (1 μl/ml) as schematically shown in Fig. 2a. For the experiments using blocking antibodies (1:100, according to the litterature), they were added to the myotubes an hour before the molecules. For fluorescence microscopy, myotubes were rinsed with PBS and fixed for 15 min at room temperature with 3% paraformaldehyde (PFA) in 0.1 M phosphate buffer, pH 7.4 and rinsed in PBS. AChR clusters were visualized using Alexa Fluor 488 or 555-conjugated α-bungarotoxin (2 μg/ml, Molecular Probes). For western blotting, total protein extracts were resolved on 10% ProSieve 50 polyacrylamide gel (FMC Bioproducts, Rockland, ME) in Tris-Tricine running buffer, transferred to PVDF membranes (Millipore), and incubated with antibodies listed in Supplemental Table 1. After the washing step of the incubation with HRP-conjugated secondary antibodies, the proteins were visualized using chemiluminescence (Amersham ECL, GE Healthcare) and quantify using ImageJ’s gel analysis. For electrophysiological recordings, HEK293 stably expressing hCaV3.2 were cultivated high glucose DMEM supplemented with 10% FBS, 1 mM L-glutamine, 1 mM penicillin/streptomycin and completed with 800 µg/ml geneticin G418.

### Electrophysiological recordings of Ca_v_3.2

For electrophysiological recordings, trypsined cells were centrifuged at 800 rpm for 4 min and resuspended (≈ 350,000 cells ml^−1^) in patch-clamp extracellular solution. Whole–cell recordings were used to investigate the effects of flunarizine on Cav3.2 expressing HEK293 cells. Automated patch-clamp recordings were performed using the SyncroPatch 384PE from Nanion (München, Germany). Chips with medium resistance single holes (n = 384) were used for electrophysiological recordings. Pulse generation and data collection were performed with the PatchControl384 v1.9.7 software (Nanion) and the Biomek interface (Beckman Coulter). Whole-cell recordings were conducted according to the recommended procedures of Nanion. Cells were stored in a cell hotel reservoir at 10 °C with shaking speed at 60 RPM. After initiating the experiment, cell catch, sealing, whole-cell formation, liquid application, recording, and data acquisition were all performed sequentially and automatically. The intracellular solution contained (in mM) : 10 CsCl, 110 CsF, 10 NaCl, 10 EGTA and 10 HEPES (pH 7.2, osmolarity 280 mOsm), and the extracellular solution contained (in mM): 60 NMDG, 80 NaCl, 4 KCl, 10 CaCl2, 1 MgCl2, 5 Glucose and 10 HEPES (pH 7.4 with NaOH). Whole-cell experiments were performed at a holding potential of −100 mV at room temperature (18–22 °C). Currents were sampled at 20 kHz. For pharmacological assays, flunarizine solutions were prepared at various concentrations in the extracellular solution supplemented with 0.3% bovine serum albumin, and distributed in 384-well compound plates according to a pre-established plate plan. This distribution within compound plates could not be randomized since it subsequently would excessively complexify the methods of analyses. The working compound solution was diluted 3 times in the patch-clamp recording well by adding 30 to 60 µL external solution to reach the final reported concentration and the test volume of 90 µL. For the control groups, vehicle solutions were extracellular containing 0.3% BSA and 0.1% DMSO (wich was similar to the DMSO concentration in the 10 µM flunarizine solution). Flunarizine effects were measured all along during a 10-min application time with a single step protocol containing a 50 ms –100 mV holding potential period followed by a 400 ms pulse at −10 mV. This single step protocol was repeated with a 10 s interval.

### Animal procedure and muscle tissue experiments

Animal experiments were performed in accordance with the ARRIVE guidelines and the protocols approved by the Université de Paris animal protection committee (CEEA 34) respecting the European and French guidelines for care and uses of laboratory animals (2010/63/EU) under the reference numbers 01246.02 and B75-06-07. The Taiwanese SMA mice were injected daily from birth with either flunarizine (500 µg/ml, 1 µl/g) or vehicle (1% DMSO in saline solution) as described^43^. The SMA mice are on FVB/NRj background (Janvier, Le Genest-St-Isle, France) and have been backcrossed for 10 generations as described. A number was assigned to each animal, and unless stated otherwise, the experiments were performed blinded for genotype, treatment and molecular and cellular studies. The group allocation was disclosed for the analyses. No exclusion criteria were used. Both females and males were included in the study. The severe Taiwanese SMA model (Smn^ko/ko^; SMN2^tg/0^) and the corresponding control heterozygous (Smn^ko/wt^; SMN2^tg/0^) mice were produced in the same litter. Based on our previous results^43^, we studied three mice per experimental group to minimize the number of animals used. The four groups were vehicle- and flunarizine-treated control and SMA model mice for a total of 59 mice. The mice were genotyped with PCR primers S1, S2, H1, 2B and 2F^131^. Briefly, a multiplex PCR reaction was set up with 1 µl DNA (20 ng), 5 µl 5X green GoTaq flexi buffer, 2.5 µl 25 mM MgCl_2_ (2.5 mM final), 1 µl 10 mM dNTPs mixture (2.5 mM each dNTP, 200 µM final), 1.5 µl 10 µM S2 forward primer (0.6 µM final), 0.75 µl 10 µM 2F forward primer (0.3 µM final), 0.75 µl 10 µM S1, H1, 2B reverse primers (0.3 µM final) and 0.15 µl GoTaq DNA polymerase (Promega). Amplification conditions were as following: 5 min at 95 °C then 30 s at 95 °C, 1 min at 58 °C, 45 s at 72 °C for 33 cycles and a final extension step of 5 min at 72 °C. The PCR products S2-S1 (1150 bp), S2-H1 (950 bp) and 2F-2B (478 bp) were analysed on a 1% agarose gel. SMA mutants and their heterozygote littermates were anesthetized by intraperitoneal injections of pentobarbital (64 mg/kg) and decapitated at P5 or P10. Three skeletal muscles (soleus, plantaris, tibialis) were dissected, snap-frozen and stored at −80 °C. The immunofluorescence experiments on muscle cryosections (10 μm) were performed as we previously detailed^43^. Briefly, the sections were fixed in 4% PFA in PBS for 20 min, blocked in 20% FCS or 4% BSA for 30 min and incubated with primary antibodies overnight at 4 °C. After 1 h in PBS-Tween (0.1%), the sections were incubated for 2 h with secondary antibodies. After bis-benzimide (or DAPI) staining and washes, the sections were mounted with fluoromount-G solution. The antibodies used are listed in Supplemental Table 1.

### Immunofluorescence microscopy image acquisition, processing and analysis

Images were taken using an ORCA Flash 2.8 camera (Hamamatsu Photonic) mounted on an epifluorescence microscope system (AxioObserver Z1, ZEISS). ZEN and ImageJ softwares were used to acquire and analyse the images, respectively. Briefly, regions of interest (ROIs) were randomly selected and manually drawn using the brightfield channel images with ImageJ. For the fluorescence intensity analyses of muscle fibers, first the threshold level was adjusted to remove any background signal and the mean gray values of Cav3.2 labelled muscle fibers were measured for fibers of either type I-labelled or –unlabelled fibers (presumably type II) for each group of mice.

The size of AChR clusters in C2C12 myotubes was determined with ImageJ by the measure of AChR aggregates (≥ 6 to 10 μm) in randomly selected 20× microscope fields (40 fields per experiment, n ≥ 3 independent experiments) or from a hundred fibers per experiment (n = 3).

### In situ proximity ligation assay

In situ proximity ligation assay (PLA) was performed as we described^132^. Briefly, C2C12 cells were fixed and permeabilized as above. Primary antibodies (Supplemental Table 1) were diluted in the antibody diluent (DuolinkII kit, Sigma-Aldrich) and incubated overnight at 4 °C. Negative controls consisted of using a single primary antibody. The cells were washed twice for 5 min in Tris-buffered saline with 0.05% Tween. The PLA probes (mouse-PLUS and rabbit-MINUS) were incubated for 1-h at 37 °C. Subsequent steps were performed using the detection reagent Orange. Signals were viewed as dots with a fluorescence microscope and analysed with ImageJ.

### RNA preparation and expression analyses

Total RNA was extracted from tissues and cell cultures using Trizol Reagent (Ambion) and treated with a RQ1 RNase-free DNase (Promega). One µg of RNA was used to generate cDNA with miScript II RT kit (Qiagen) for snRNA analyses and Superscript III (Invitrogen) for the other genes. Quantitative real-time PCR was performed in triplicate using the primers listed in Supplemental Table 2 with SYBR Green ROX mix (Thermo Scientific) on either Applied Biosystems 7500 fast system or BioRad CFX384. The normalized expression levels were calculated according to the ΔΔCt method. The snRNA, 5 S, 5.8 S, *Rpl13a, Ppia, Gapdh, Myh4* and Z^+^ *Agrn* primers and their analyses have been previously described^43^. Additional primers have been designed using the free primer design tools from eurofins (eurofinsgenomics.eu) and validated according to the MIQE guidelines.

### Statistical analyses

At least three independent experiments were presented as the mean ± SD or SEM. Statistical analyses were conducted using the non-parametric Kruskal Wallis followed by Dunn’s multiple comparison rank test or two-way ANOVA followed by Tukey’s multiple comparisons test or Student t’test (GraphPad). The test used was indicated in the figure legends. The significant threshold was 5%.

## Supplementary Information

Supplementary Information.

## Data Availability

The detailed calculations made during and/or analysed during the current study are available from the corresponding author on reasonable request.

## References

[1] Lefebvre S (1995). Identification and characterization of a spinal muscular atrophy-determining gene. Cell.

[2] Lefebvre S (1997). Correlation between severity and SMN protein level in spinal muscular atrophy. Nat. Genet..

[3] Cauchi RJ (2010). SMN and Gemins: “We are family” or are we? Insights into the partnership between Gemins and the spinal muscular atrophy disease protein SMN. BioEssays.

[4] Singh RN, Howell MD, Ottesen EW, Singh NN (2017). Diverse role of survival motor neuron protein. Biochim. Biophys. Acta Gene Regul. Mech..

[5] So, B.R., Zhang, Z. & Dreyfuss, G. The SMN complex: Organization and function. in *Spinal Muscular Atrophy: Disease Mechanisms and Therapy* (Sumner, C.J., Paushkin, S., Ko, C.P. eds.). (Academic Press, 2016).

[6] Lorson CL, Androphy EJ (2000). An exonic enhancer is required for inclusion of an essential exon in the SMA-determining gene SMN. Hum. Mol. Genet..

[7] Burghes AHM, Beattie CE (2009). Spinal muscular atrophy: Why do low levels of survival motor neuron protein make motor neurons sick?. Nat. Rev. Neurosci..

[8] Calucho M (2018). Correlation between SMA type and SMN2 copy number revisited: An analysis of 625 unrelated Spanish patients and a compilation of 2834 reported cases. Neuromuscul. Disord..

[9] Finkel RS, Fishbeck KH (2021). Maybe too much of a good thing in gene therapy. Nat. Neurosci..

[10] Groen EJN, Talbot K, Gillingwater TH (2018). Advances in therapy for spinal muscular atrophy: Promises and challenges. Nat. Rev. Neurol..

[11] Darras BT (2021). Risdiplam-Treated Infants with type 1 spinal muscular atrophy versus historical controls. N. Engl. J. Med..

[12] Mercuri E, Pera MC, Scoto M, Finkel R, Muntoni F (2020). Spinal muscular atrophy—Insights and challenges in the treatment era. Nat. Rev. Neurol..

[13] Ojala KS, Reedich EJ, DiDonato CJ, Meriney SD (2021). In search of a cure: The development of therapeutics to alter the progression of spinal muscular atrophy. Brain Sci..

[14] Braun S, Croizat B, Lagrange MC, Warter JM, Poindron P (1995). Constitutive muscular abnormalities in culture in spinal muscular atrophy. Lancet.

[15] Cifuentes-Diaz C (2002). Neurofilament accumulation at the motor endplate and lack of axonal sprouting in a spinal muscular atrophy mouse model. Hum. Mol. Genet..

[16] Martínez-Hernández R, Bernal S, Alias L, Tizzano EF (2014). Abnormalities in early markers of muscle involvement support a delay in myogenesis in spinal muscular atrophy. J. Neuropathol. Exp. Neurol..

[17] Biondi O (2008). Exercise-induced activation of NMDA receptor promotes motor unit development and survival in a type 2 spinal muscular atrophy model mouse. J. Neurosci..

[18] Kariya S (2008). Reduced SMN protein impairs maturation of the neuromuscular junctions in mouse models of spinal muscular atrophy. Hum. Mol. Genet..

[19] Kong L (2009). Impaired synaptic vesicle release and immaturity of neuromuscular junctions in spinal muscular atrophy mice. J. Neurosci..

[20] Sanes JR, Lichtman JW (2001). Induction, assembly, maturation and maintenance of a postsynaptic apparatus. Nat. Rev. Neurosci..

[21] Nishimune H, Shigemoto K (2018). Practical anatomy of the neuromuscular junction in health and disease. Neurol. Clin..

[22] Li L, Xiong WC, Mei L (2018). Neuromuscular junction formation, aging, and disorders. Annu. Rev. Physiol..

[23] Burgess RW, Nguyen QT, Son YJ, Lichtman JW, Sanes JR (1999). Alternatively spliced isoforms of nerve- and muscle-derived agrin: their roles at the neuromuscular junction. Neuron.

[24] Kröger S, Schröder JE (2002). Agrin in the developing CNS: New roles for a synapse organizer. News Physiol. Sci..

[25] Tintignac LA, Brenner HR, Rüegg MA (2015). Mechanisms regulating neuromuscular junction development and function and causes of muscle wasting. Physiol. Rev..

[26] Ruegg MA, Bixby JL (1998). Agrin orchestrates synaptic differentiation at the vertebrate neuromuscular junction. Trends Neurosci..

[27] Bezakova G, Ruegg MA (2003). New insights into the roles of agrin. Nat. Rev. Mol. Cell. Biol..

[28] Burgess RW, Dickman DK, Nunez L, Glass DJ, Sanes JR (2002). Mapping sites responsible for interactions of agrin with neurons. J. Neurochem..

[29] Tezuka T (2014). The MuSK activator agrin has a separate role essential for postnatal maintenance of neuromuscular synapses. Proc. Natl. Acad. Sci. USA.

[30] Bassat E (2017). The extracellular matrix protein agrin promotes heart regeneration in mice. Nature.

[31] Campagna JA, Rüegg MA, Bixby JL (1995). Agrin is a differentiation-inducing "stop signal" for motoneurons in vitro. Neuron.

[32] Chang D, Woo JS, Campanelli J, Scheller RH, Ignatius MJ (1997). Agrin inhibits neurite outgrowth but promotes attachment of embryonic motor and sensory neurons. Dev. Biol..

[33] Bixby JL, Baerwald-De la Torre K, Wang C, Rathjen FG, Rüegg MA (2002). A neuronal inhibitory domain in the N-terminal half of agrin. J. Neurobiol..

[34] Handara G, Kröger S (2019). Alternative splicing and the intracellular domain mediate TM-agrin's ability to differentially regulate the density of excitatory and inhibitory synapse-like specializations in developing CNS neurons. Neuroscience.

[35] Chakraborty S (2015). An oncogenic role of Agrin in regulating focal adhesion integrity in hepatocellular carcinoma. Nat. Commun..

[36] Chakraborty S, Njah K, Hong W (2020). Agrin mediates angiogenesis in the tumor microenvironment. Trends Cancer.

[37] Zhang Z (2013). Dysregulation of synaptogenesis genes antecedes motor neuron pathology in spinal muscular atrophy. Proc. Natl. Acad. Sci. USA.

[38] Tisdale S, Van Alstyne M, Simon CM, Mentis GZ, Pellizzoni L (2022). SMN controls neuromuscular junction integrity through U7 snRNP. Cell Rep..

[39] Kim JK, Caine C, Awano T, Herbst R, Monani UR (2017). Motor neuronal repletion of the NMJ organizer, Agrin, modulates the severity of the spinal muscular atrophy disease phenotype in model mice. Hum. Mol. Genet..

[40] Boido M (2018). Increasing agrin function antagonizes muscle atrophy and motor impairment in spinal muscular atrophy. Front. Cell. Neurosci..

[41] Kaifer KA (2020). AAV9-DOK7 gene therapy reduces disease severity in Smn 2B/- SMA model mice. Biochem. Biophys. Res. Commun..

[42] Feng Z (2021). Activation of muscle-specific kinase (MuSK) reduces neuromuscular defects in the delta7 mouse model of spinal muscular atrophy (SMA). Int. J. Mol. Sci..

[43] Sapaly D (2018). Small-molecule flunarizine increases SMN protein in nuclear Cajal bodies and motor function in a mouse model of spinal muscular atrophy. Sci. Rep..

[44] Santi CM (2002). Differential inhibition of T-type calcium channels by 422 neuroleptics. Neuroscience.

[45] Younis I, Berg M, Kaida D, Dittmar K, Wang C, Dreyfuss G (2010). Rapid-response splicing reporter screens identify differential regulators of constitutive and alternative splicing. Mol. Cell. Biol..

[46] Bäumer D (2009). Alternative splicing events are a late feature of pathology in a mouse model of spinal muscular atrophy. PLoS Genet..

[47] Doktor TK (2017). RNA-sequencing of a mouse-model of spinal muscular atrophy reveals tissue-wide changes in splicing of U12-dependent introns. Nucleic Acids Res..

[48] Jablonka S, Beck M, Lechner BD, Mayer C, Sendtner M (2007). Defective Ca^2+^ channel clustering in axon terminals disturbs excitability in motoneurons in spinal muscular atrophy. J. Cell Biol..

[49] Ruiz R, Casañas JJ, Torres-Benito L, Cano R, Tabares L (2010). Altered intracellular Ca^2+^ homeostasis in nerve terminals of severe spinal muscular atrophy mice. J. Neurosci..

[50] Wirth B (2021). Spinal muscular atrophy: In the challenge lies a solution. Trends Neurosci..

[51] Zhang Z (2008). SMN deficiency causes tissue-specific perturbations in the repertoire of snRNAs and widespread defects in splicing. Cell.

[52] Zhu D, Yang Z, Luo Z, Luo S, Xiong WC, Mei L (2008). Muscle-specific receptor tyrosine kinase endocytosis in acetylcholine receptor clustering in response to agrin. J. Neurosci..

[53] Lin SS (2020). Dynamin-2 regulates postsynaptic cytoskeleton organization and neuromuscular junction development. Cell Rep..

[54] Chen PJ, Zelada D, Belhasan DC, Akaaboune M (2020). Phosphorylation of α-dystrobrevin is essential for αkap accumulation and acetylcholine receptor stability. J. Biol. Chem..

[55] Martinez-Pena y Valenzuela I, Mouslim C, Akaaboune M (2010). Calcium/calmodulin kinase II-dependent acetylcholine receptor cycling at the mammalian neuromuscular junction in vivo. J. Neurosci..

[56] Sapaly D, Delers P, Coridon J, Salman B, Letourneur F, Dumont F, Lefebvre S (2020). The small-molecule flunarizine in spinal muscular atrophy patient fibroblasts impacts on the gemin components of the SMN complex and TDP43, an RNA-binding protein relevant to motor neuron diseases. Front. Mol. Biosci..

[57] Martin PT, Sanes JR (1997). Integrins mediate adhesion to agrin and modulate agrin signalling. Development.

[58] Gee SH, Montanro F, Lindenbaum MH, Carbonetto S (1994). Dystroglycan-a, a dystrophin-associated glycoprotein, is a functional agrin receptor. Cell.

[59] Jacobson, C., Côté, P.D., Rossi, S.G., Rotundo, R.L. & Carbonetto, S. The dystroglycan complex is necessary for stabilization of acetylcholine receptor clusters at neuromuscular junctions and formation of the synaptic basement membrane. *J. Cell Biol.* **152**, 435–450 (2001). 10.1083/jcb.152.3.435 PMC2195998 11157973

[60] Campanelli JT, Hoch W, Rupp F, Kreiner T, Scheller RH (1991). Agrin mediates cell contact-induced acetylcholine receptor clustering. Cell.

[61] Patel AA, Steitz JA (2003). Splicing double: Insights from the second spliceosome. Nat. Rev. Mol. Cell. Biol..

[62] Osman EY (2020). Minor snRNA gene delivery improves the loss of proprioceptive synapses on SMA motor neurons. JCI Insight.

[63] Gonoi T, Hasegawa S (1988). Post-natal disappearance of transient calcium channels in mouse skeletal muscle: Effects of denervation and culture. J. Physiol..

[64] Canto-Bustos M, Loeza-Alcocer E, González-Ramírez R (2014). Functional expression of T-type Ca^2+^ channels in spinal motoneurons of the adult turtle. PLoS ONE.

[65] Zhang Z, David G (2016). Stimulation-induced Ca(2+) influx at nodes of Ranvier in mouse peripheral motor axons. J. Physiol..

[66] Bernal Sierra YA, Haseleu J, Kozlenkov A (2017). Genetic Tracing of Cav3.2 T-type calcium channel expression in the peripheral nervous system. Front. Mol. Neurosci..

[67] Braunstein TH, Inoue R, Cribbs L (2009). Calcium channels in local and remote calcium responses in rat mesenteric terminal arterioles. J. Vasc. Res..

[68] Pollari E, Goldsteins G, Bart G, Koistinaho J, Giniatullin R (2014). The role of oxidative stress in degeneration of the neuromuscular junction in amyotrophic lateral sclerosis. Front. Cell. Neurosci..

[69] Xu G, Chen J, Jing G, Shalev A (2012). Preventing β-cell loss and diabetes with calcium channel blockers. Diabetes.

[70] Wen Y (2021). Myonuclear transcriptional dynamics in response to exercise following satellite cell depletion. iScience.

[71] Hayhurst M, Wagner AK, Cerletti M, Wagers AJ, Rubin LL (2012). A cell-autonomous defect in skeletal muscle satellite cells expressing low levels of survival of motor neuron protein. Dev. Biol..

[72] Mecca J, Astord S, Marais T, Relaix F, Didier N, Barkats M (2017). Role of muscle satellite cells in Spinal muscular atrophy physiopathology. Neuromusc. Disord..

[73] Nicole S (2003). Intact satellite cells lead to remarkable protection against Smn gene defect in differentiated skeletal muscle. J. Cell. Biol..

[74] Kawamoto E, Tamakoshi K, Ra SG, Masuda H, Kawanaka K (2018). Immobilization rapidly induces thioredoxin-interacting protein gene expression together with insulin resistance in rat skeletal muscle. J. Appl. Physiol..

[75] Murray LM, Talbot K, Gillingwater TH (2010). Review: Neuromuscular synaptic vulnerability in motor neurone disease: Amyotrophic lateral sclerosis and spinal muscular atrophy. Neuropathol. Appl. Neurobiol..

[76] Gromova A, La Spada AR (2020). Harmony lost: Cell–cell communication at the neuromuscular junction in motor neuron disease. Trends Neurosci..

[77] Menti GZ (2011). Early functional impairment of sensory-motor connectivity in a mouse model of spinal muscular atrophy. Neuron.

[78] Fletcher EV (2017). Reduced sensory synaptic excitation impairs motor neuron function via Kv2.1 in spinal muscular atrophy. Nat. Neurosci..

[79] Vukojicic A (2019). The classical complement pathway mediates microglia-dependent remodeling of spinal motor circuits during development and in SMA. Cell Rep..

[80] Monani UR (2000). The human centromeric survival motor neuron gene (SMN2) rescues embryonic lethality in Smn(−/−) mice and results in a mouse with spinal muscular atrophy. Hum. Mol. Genet..

[81] Buettner JM (2021). Central synaptopathy is the most conserved feature of motor circuit pathology across spinal muscular atrophy mouse models. iScience.

[82] Shi L, Fu AKY, Ip N (2012). Molecular mechanisms underlying maturation and maintenance of the vertebrate neuromuscular junction. Trends Neurosci..

[83] Besse A (2020). AAV9-mediated expression of SMN restricted to neurons does not rescue the spinal muscular atrophy phenotype in mice. Mol. Ther..

[84] Kim JK, Monani UR (2018). Augmenting the SMN protein to treat infantile spinal muscular atrophy. Neuron.

[85] Tisdale, S. & Pellizzoni, L. RNA-processing dysfunction in spinal muscular atrophy. in *Spinal Muscular Atrophy: Disease Mechanisms and Therapy* (Sumner, C.J., Paushkin, S., Ko, C.P. eds.) (Academic Press, 2016).

[86] Muntoni F (2020). Long-term follow-up of patients with type 2 and non-ambulant type 3 spinal muscular atrophy (SMA) treated with olesoxime in the OLEOS trial. Neuromuscul. Disord..

[87] Chaytow H, Faller KME, Huang YT, Gillingwater TH (2021). Spinal muscular atrophy: From approved therapies to future therapeutic targets for personalized medicine. Cell Rep. Med..

[88] Meijboom KE (2021). Combining multiomics and drug perturbation profiles to identify muscle-specific treatments for spinal muscular atrophy. JCI Insight.

[89] Rodríguez Cruz PM, Cossins J, Beeson D, Vincent A (2020). The neuromuscular junction in health and disease: Molecular mechanisms governing synaptic formation and homeostasis. Front. Mol. Neurosci..

[90] Gesemann M, Cavalli V, Denzer AJ, Brancaccio A, Schumacher B, Ruegg MA (1996). Alternative splicing of agrin alters its binding to heparin, dystroglycan, and the putative agrin receptor. Neuron.

[91] Meier T, Ruegg MA, Wallace BG (1998). Muscle-specific agrin isoforms reduce phosphorylation of AChR gamma and delta subunits in cultured muscle cells. Mol. Cell Neurosci..

[92] Jones G, Herczeg A, Ruegg MA, Lichtsteiner M, Kröger S, Brenner HR (1996). Substrate-bound agrin induces expression of acetylcholine receptor epsilon-subunit gene in cultured mammalian muscle cells. Proc. Natl. Acad. Sci. USA.

[93] Meier T (1998). Agrin can mediate acetylcholine receptor gene expression in muscle by aggregation of muscle-derived neuregulins. J. Cell Biol..

[94] Ferns M, Hoch W, Campanelli JT, Rupp F, Hall ZW, Scheller RH (1992). RNA splicing regulates agrin-mediated acetylcholine receptor clustering activity on cultured myotubes. Neuron.

[95] Khan AA, Bose C, Yam LS, Soloski MJ, Rupp F (2001). Physiological regulation of the immunological synapse by agrin. Science.

[96] Bowe MA, Deyst KA, Leszyk JD, Fallon JR (1994). Identification and purification of an agrin receptor from Torpedo postsynaptic membranes: A heteromeric complex related to the dystroglycans. Neuron.

[97] Burg MA, Halfter W, Cole GJ (1995). Analysis of proteoglycan expression in developing chicken brain: Characterization of a heparan sulfate proteoglycan that interacts with the neural cell adhesion molecule. J. Neurosci. Res..

[98] Denzer AJ, Brandenberger R, Gesemann M, Chiquet M, Ruegg MA (1997). Agrin binds to the nerve-muscle basal lamina via laminin. J. Cell. Biol..

[99] Sugiyama J, Bowen DC, Hall ZW (1994). Dystroglycan binds nerve and muscle agrin. Neuron.

[100] Rossoll W (2003). Smn, the spinal muscular atrophy-determining gene product, modulates axon growth and localization of beta-actin mRNA in growth cones of motoneurons. J. Cell. Biol..

[101] Alkaslasi MR, Piccus ZE, Hareendran S (2021). Single nucleus RNA-sequencing defines unexpected diversity of cholinergic neuron types in the adult mouse spinal cord. Nat. Commun..

[102] Carter MT, McMillan HJ, Tomin A, Weiss N (2019). Compound heterozygous CACNA1H mutations associated with severe congenital amyotrophy. Channels.

[103] Rzhepetskyy Y, Lazniewska J, Blesneac I, Pamphlett R, Weiss N (2016). CACNA1H missense mutations associated with amyotrophic lateral sclerosis alter Cav3.2 T-type calcium channel activity and reticular thalamic neuron firing. Channels (Austin).

[104] Steinberg KM, Yu B, Koboldt DC, Mardis ER, Pamphlett R (2015). Exome sequencing of case-unaffected-parents trios reveals recessive and de novo genetic variants in sporadic ALS. Sci. Rep..

[105] Stringer RN (2020). A rare CACNA1H variant associated with amyotrophic lateral sclerosis causes complete loss of Cav3.2 T-type channel activity. Mol. Brain.

[106] Kaplan MM (2018). Calcium influx and release cooperatively regulate AChR patterning and motor axon outgrowth during neuromuscular junction formation. Cell Rep..

[107] Perez-Reyes E (2003). Molecular physiology of low-voltage-activated T-type calcium channels. Physiol. Rev..

[108] Nussbacher JK, Tabet R, Yeo GW, Lagier-Tourenne C (2019). Disruption of RNA metabolism in neurological diseases and emerging therapeutic interventions. Neuron.

[109] Weiss N, Zamponi GW (2020). Genetic T-type calcium channelopathies. J. Med. Genet..

[110] Wallace BG (1988). Regulation of agrin-induced acetylcholine receptor aggregation by Ca^2+^ and phorbol ester. J. Cell Biol..

[111] Megeath LJ, Fallon JR (1998). Intracellular calcium regulates agrin-induced acetylcholine receptor clustering. J. Neurosci..

[112] Buckingham M (2001). Skeletal muscle formation in vertebrates. Curr. Opin. Genet. Dev..

[113] Bijlenga P, Liu JH, Espinos E, Haenggeli CA, Fischer-Lougheed J, Bader CR, Bernheim L (2000). T-type α1H Ca^2+^ channels are involved in Ca^2+^ signaling during terminal differentiation (fusion) of human myoblasts. Proc. Natl. Acad. Sci. USA.

[114] Schiaffino S, Reggiani C (2011). Fiber types in mammalian skeletal muscles. Physiol. Rev..

[115] Ono K, Iijima T (2010). Cardiac T-type Ca^2+^ channels in the heart. J. Mol. Cell. Cardiol..

[116] Kumar M (2020). ELM—The eukaryotic linear motif resource in 2020. Nucleic Acids Res..

[117] Wang S, Cortes CJ (2021). Interactions with PDZ proteins diversify voltage-gated calcium channel signalling. J. Neurosci. Res..

[118] Brenman JE (1996). Interaction of nitric oxide synthase with the postsynaptic density protein PSD-95 and alpha1-syntrophin mediated by PDZ domains. Cell.

[119] Adams ME, Anderson KN, Froehner SC (2010). The alpha-syntrophin PH and PDZ domains scaffold acetylcholine receptors, utrophin, and neuronal nitric oxide synthase at the neuromuscular junction. J. Neurosci..

[120] Strochlic L, Cartaud A, Labas V, Hoch W, Rossier J, Cartaud J (2001). MAGI-1c: A synaptic MAGUK interacting with muSK at the vertebrate neuromuscular junction. J. Cell Biol..

[121] Lu Z, Je HS, Young P, Gross J, Lu B, Feng G (2007). Regulation of synaptic growth and maturation by a synapse-associated E3 ubiquitin ligase at the neuromuscular junction. J. Cell Biol..

[122] Mulatz, K.J. *A PDZ-3 Mediated Physical and Functional Interaction Between the Cav3.2 T-type Calcium Channel and Neuronal Nitric Oxide Synthase*. Doctoral Dissertation. (University of British, 2013).

[123] Grozdanovic Z, Christova T, Gossrau R (1997). Differences in the localization of the postsynaptic nitric oxide synthase I and acetylcholinesterase suggest a heterogeneity of neuromuscular junctions in rat and mouse skeletal muscles. Acta Histochem..

[124] Finanger Hedderick EL (2011). Loss of sarcolemmal nNOS is common in acquired and inherited neuromuscular disorders. Neurology.

[125] Tarabal O (2014). Mechanisms involved in spinal cord central synapse loss in a mouse model of spinal muscular atrophy. J. Neuropathol. Exp. Neurol..

[126] Clementi E (1998). Role of nitric oxide and its intracellular signalling pathways in the control of Ca^2+^ homeostasis. Biochem. Pharmacol..

[127] Shen C, Li L, Zhao K (2018). Motoneuron Wnts regulate neuromuscular junction development. Elife.

[128] Xx Motyl AAL (2020). Pre-natal manifestation of systemic developmental abnormalities in spinal muscular atrophy. Hum. Mol. Genet..

[129] Olaso R (2006). Activation of RNA metabolism-related genes in mouse but not human tissues deficient in SMN. Physiol. Genomics.

[130] Millino C (2009). Different atrophy-hypertrophy transcription pathways in muscles affected by severe and mild spinal muscular atrophy. BMC Med..

[131] Hsieh-Li HM (2000). A mouse model for spinal muscular atrophy. Nat. Genet..

[132] Renvoisé B, Quérol G, Verrier ER, Burlet P, Lefebvre S (2012). A role for protein phosphatase PP1γ in SMN complex formation and subnuclear localization to Cajal bodies. J. Cell Sci..

