# Supplementary material for: A link between agrin signalling and Cav3.2 at the neuromuscular junction in spinal muscular atrophy
Source: Sci Rep. 2022 Nov 8;12:18960. doi: 10.1038/s41598-022-23703-x (PMC9643518; doi:10.1038/s41598-022-23703-x)
Supplement: Supplementary file 1 — Supplementary Information. [file 41598_2022_23703_MOESM1_ESM.pdf]

Online supplemental material

A link between agrin signalling and $Ca_v3.2$ at the neuromuscular junction in spinal muscular atrophy

Perrine Delers, Delphine Sapaly, Badih Salman, Stéphan de Waard, Michel de Waard and Suzie Lefebvre*

* corresponding author: suzie.lefebvre@inserm.fr

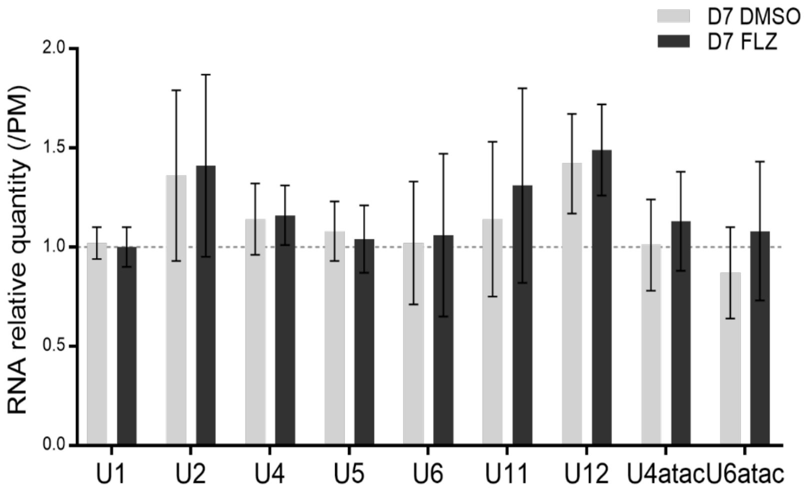

Supplemental Figure 1. Effects of flunarizine on the expression of major and minor snRNAs in murine C2C12 myotubes. The snRNA levels are determined by RT-qPCR and the relative amount is presented as fold-change of the flunarizine (Fz) treatment compared to DMSO (arbitrary unit of 1). The 5 S and 5.8 S are used as controls for normalization as described previously (Sapaly et al., 2018). Error bars indicate the S.E.M (three independent experiments, Student's t-test, not significant).

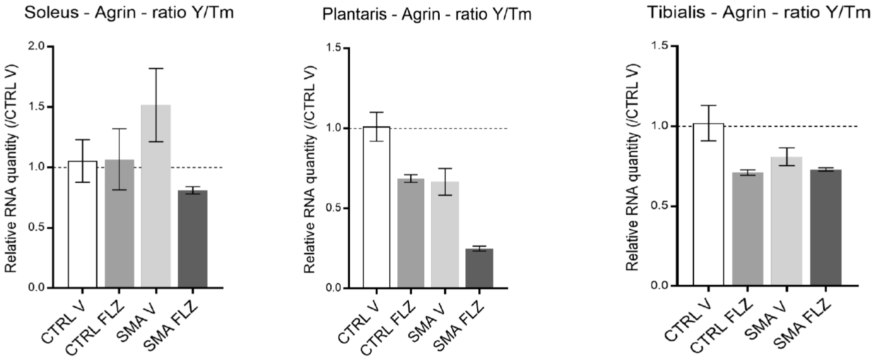

Supplemental Figure 2. Effects of flunarizine on the proportion of Y exon relative to T m exon levels in skeletal muscles of SMA mice. The exon levels determined by RT-qPCR (in Fig. 2) are used to calculate the relative Y to Tm RNA levels. (3 mice per group).

Supplemental Figure 3. C2C12 myotubes were treated by DMSO or flunarizine for overnight. Protein levels were evaluated by western blotting and analysed with ImageJ gels tool. The figure S3 presents the full-length immunoblots and images of the five Fig.1F panels and of Fig. 3D. There are three independent experiments on each blot and the selection of a panel is marked by a rectangular shape according to the mean value of protein levels from 3 to 10 independent experiments. The same blot was used only for SMN, GAPDH (Fig. 1F fourth panel) and Cav3.2 (Fig.3D) but the cropped images were not from the same experiment (8 pages).

Fig. 1F (first panel)

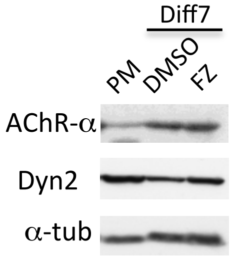

The images of the original blot

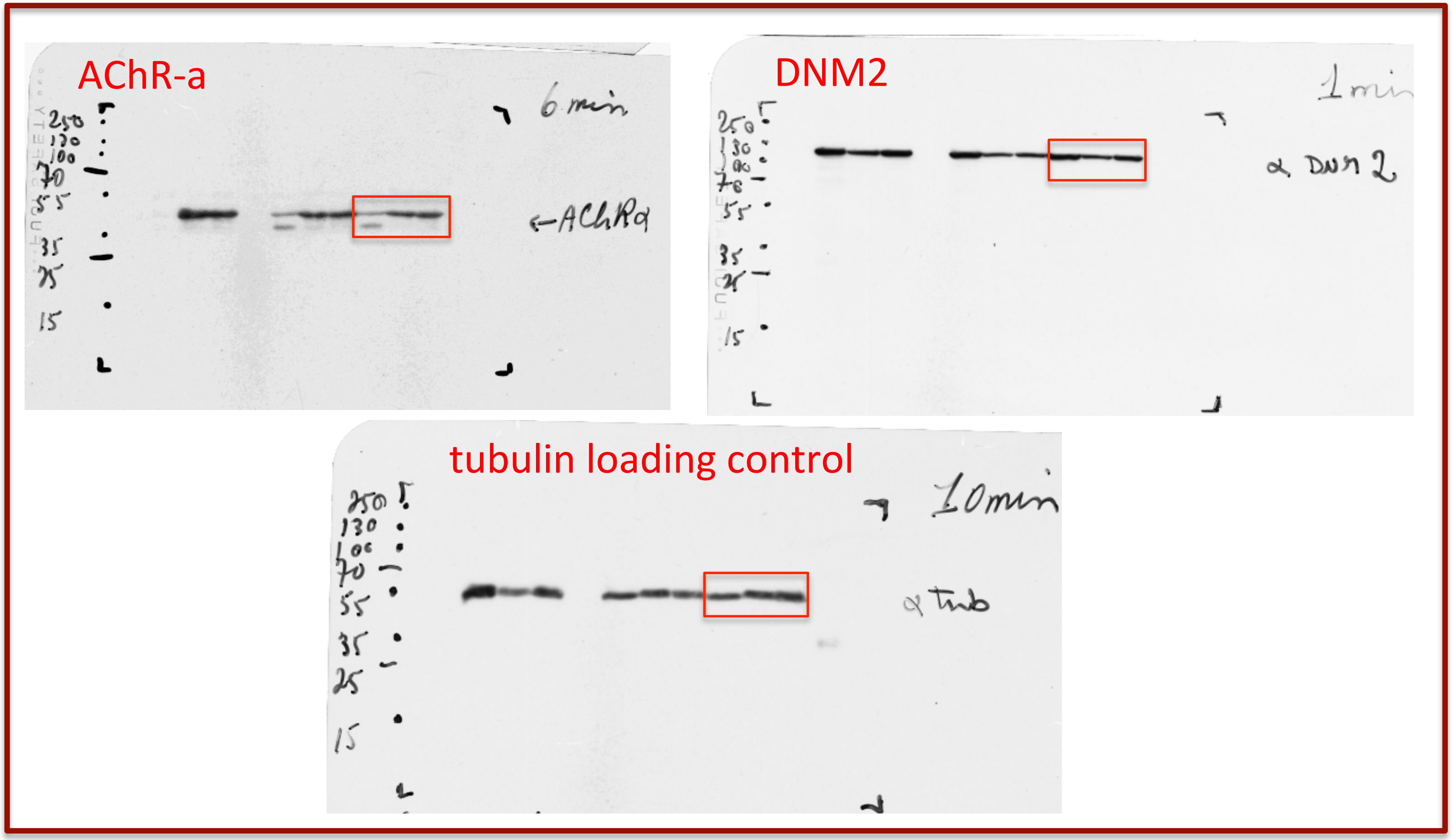

Fig. 1F (second panel)

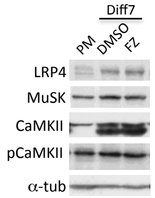

The images of the original blot

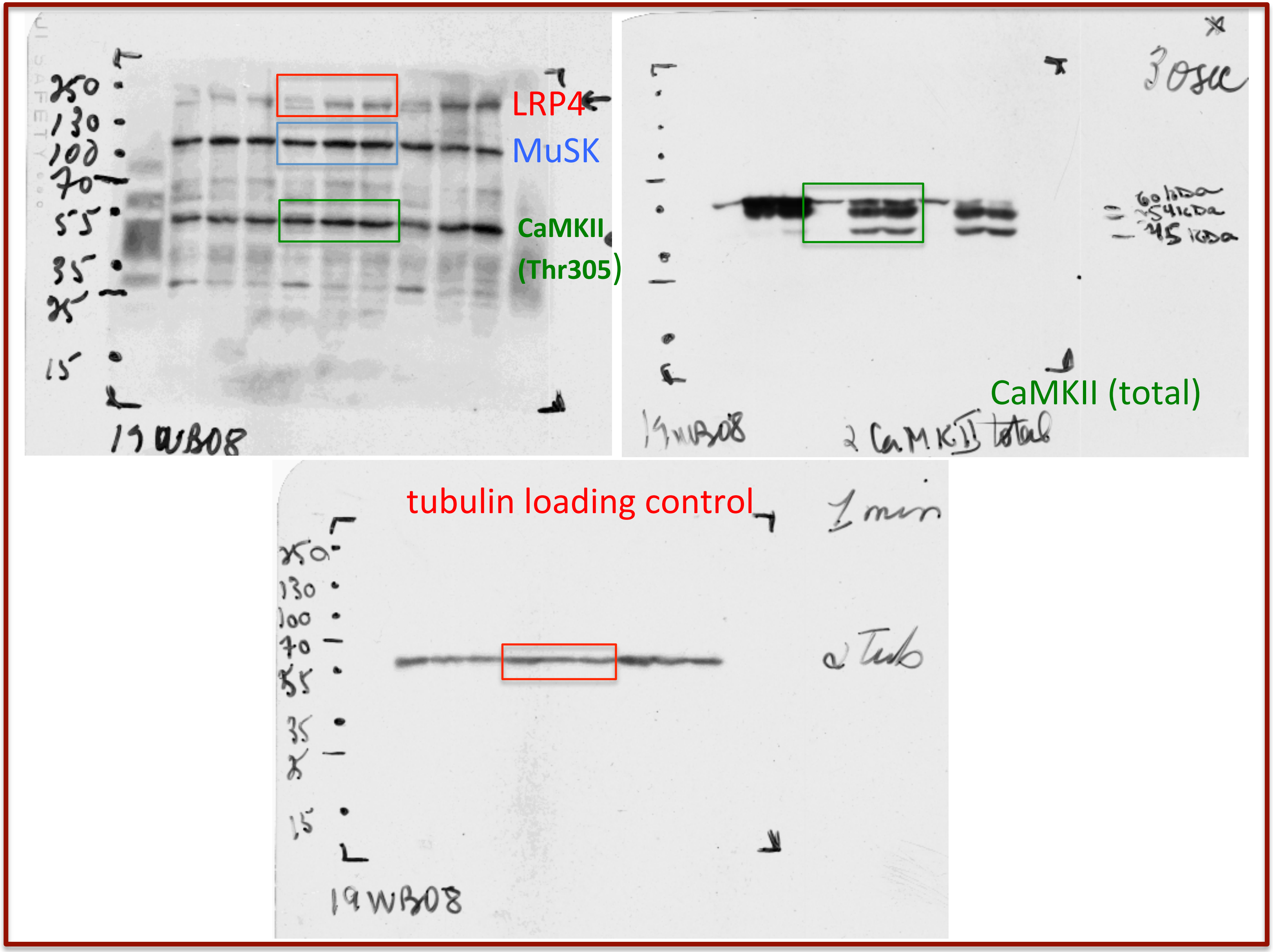

Fig. 1F (third panel)

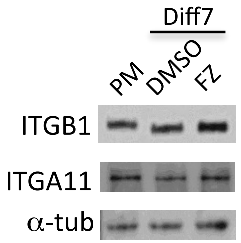

The images of the original blot

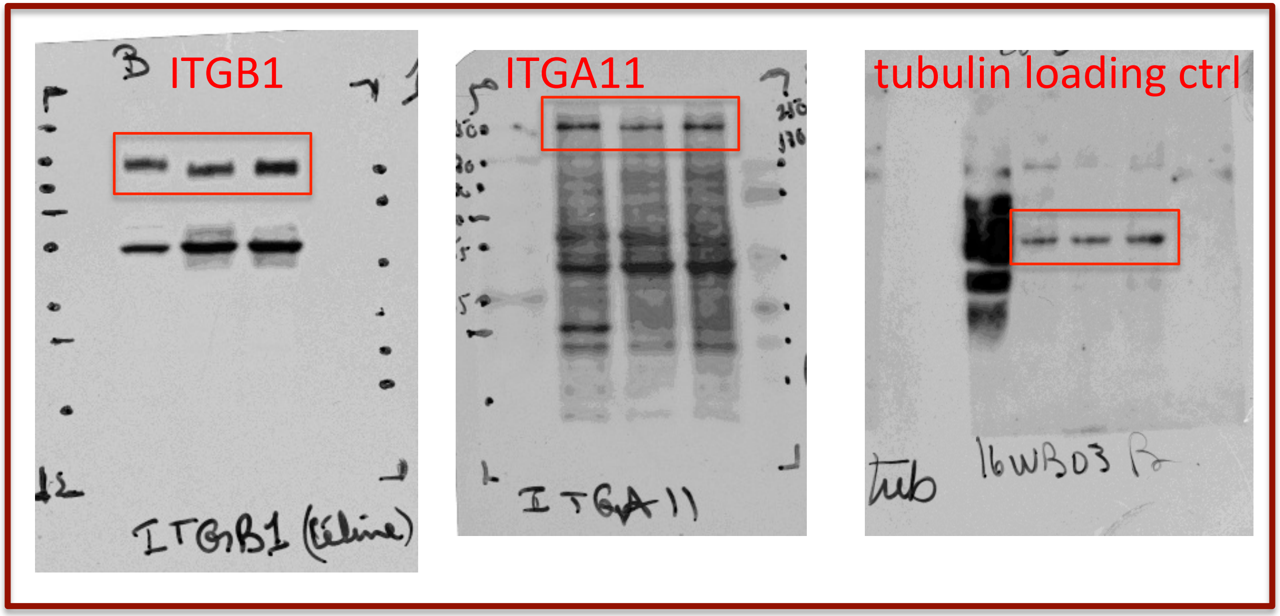

Fig. 1F (fourth panel)

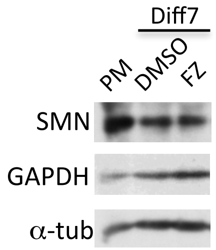

The images of the original blot

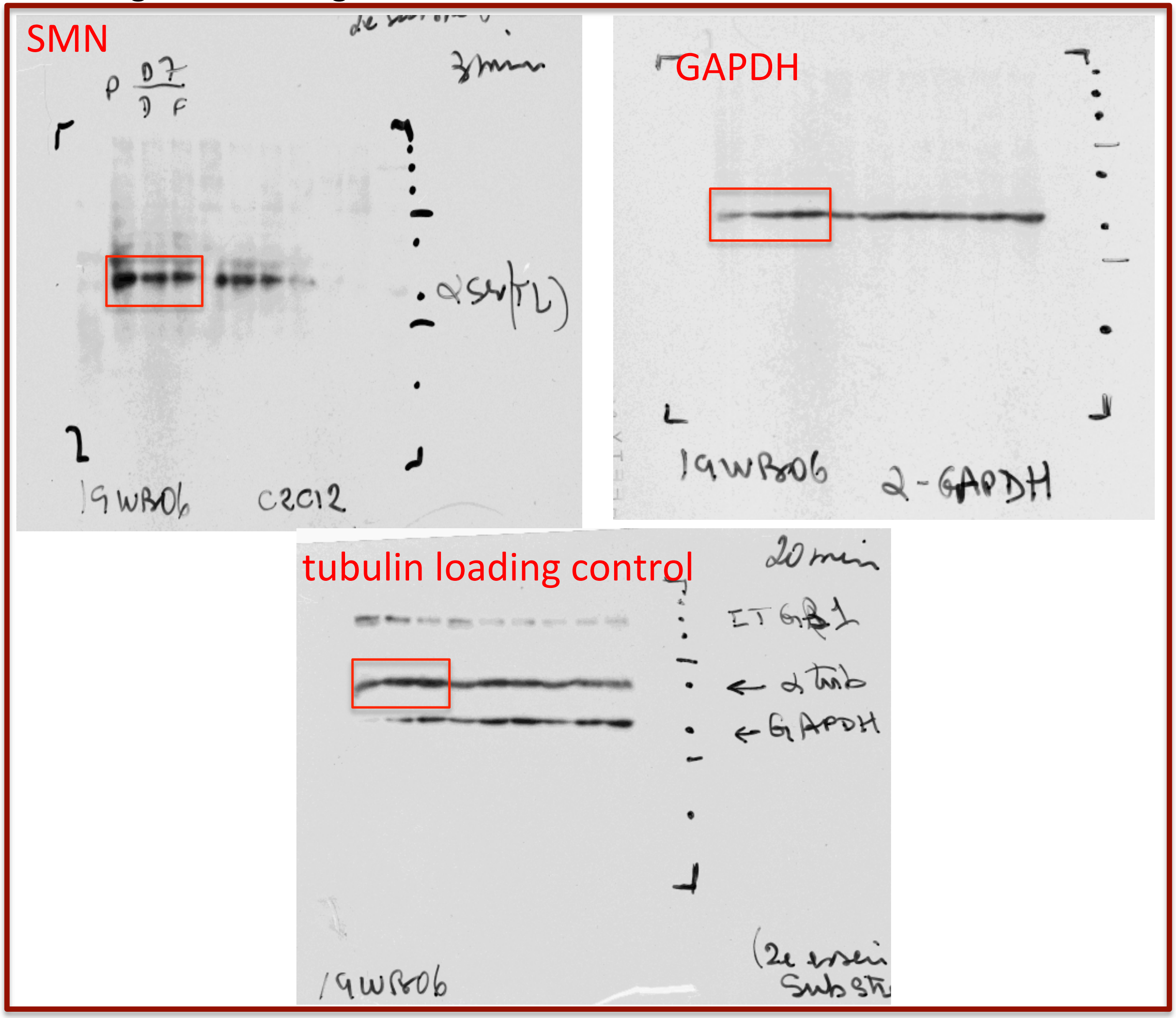

Fig. 1F (fifth panel)

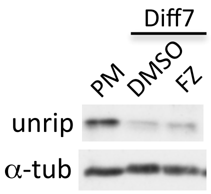

The images of the original blot

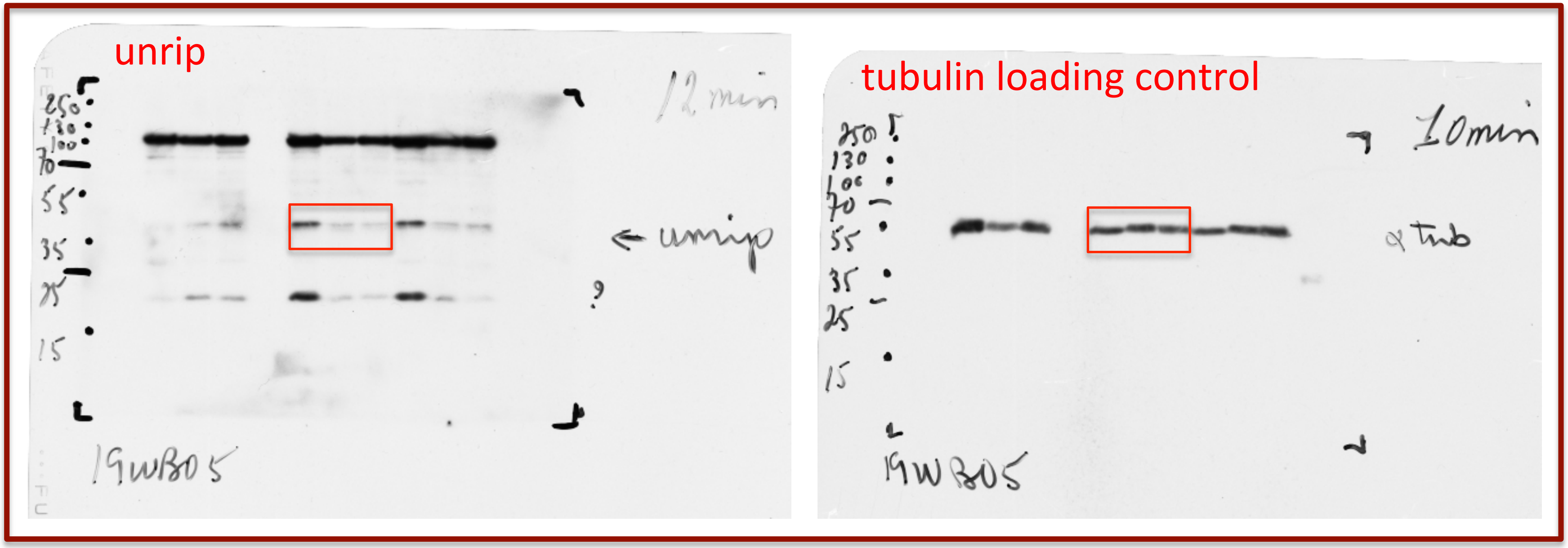

Fig. 1F (last panel)

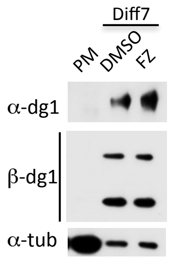

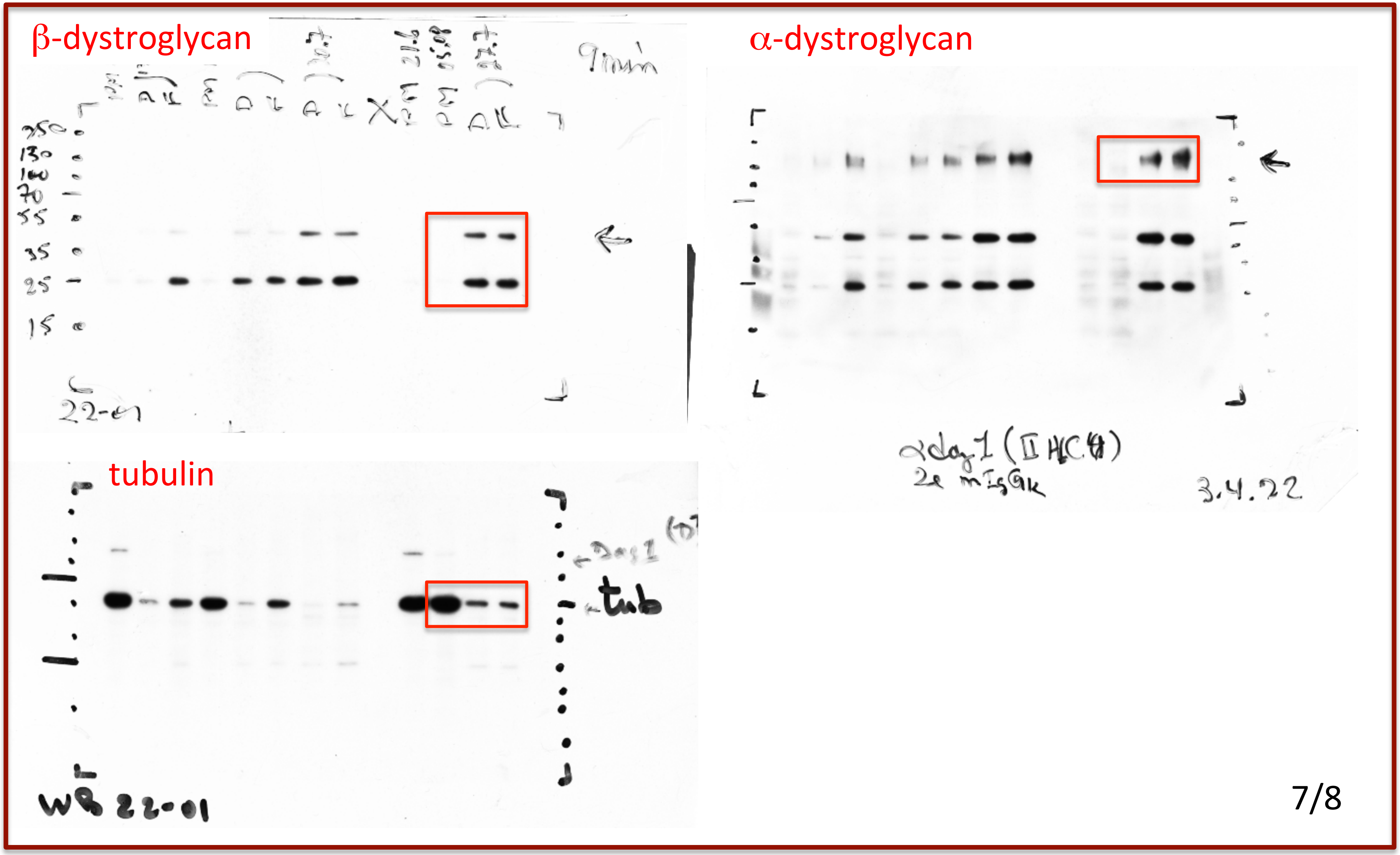

Fig. 3D

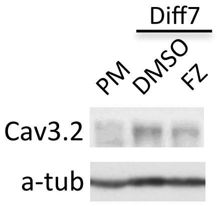

The images of the original blot

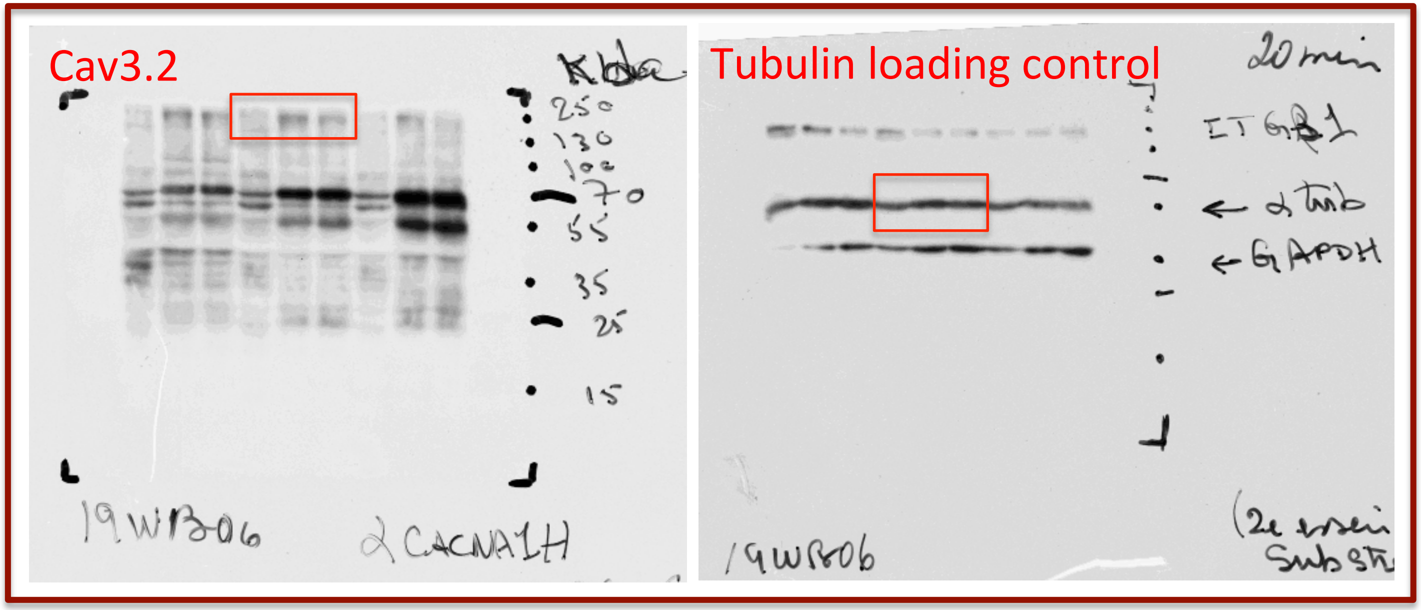

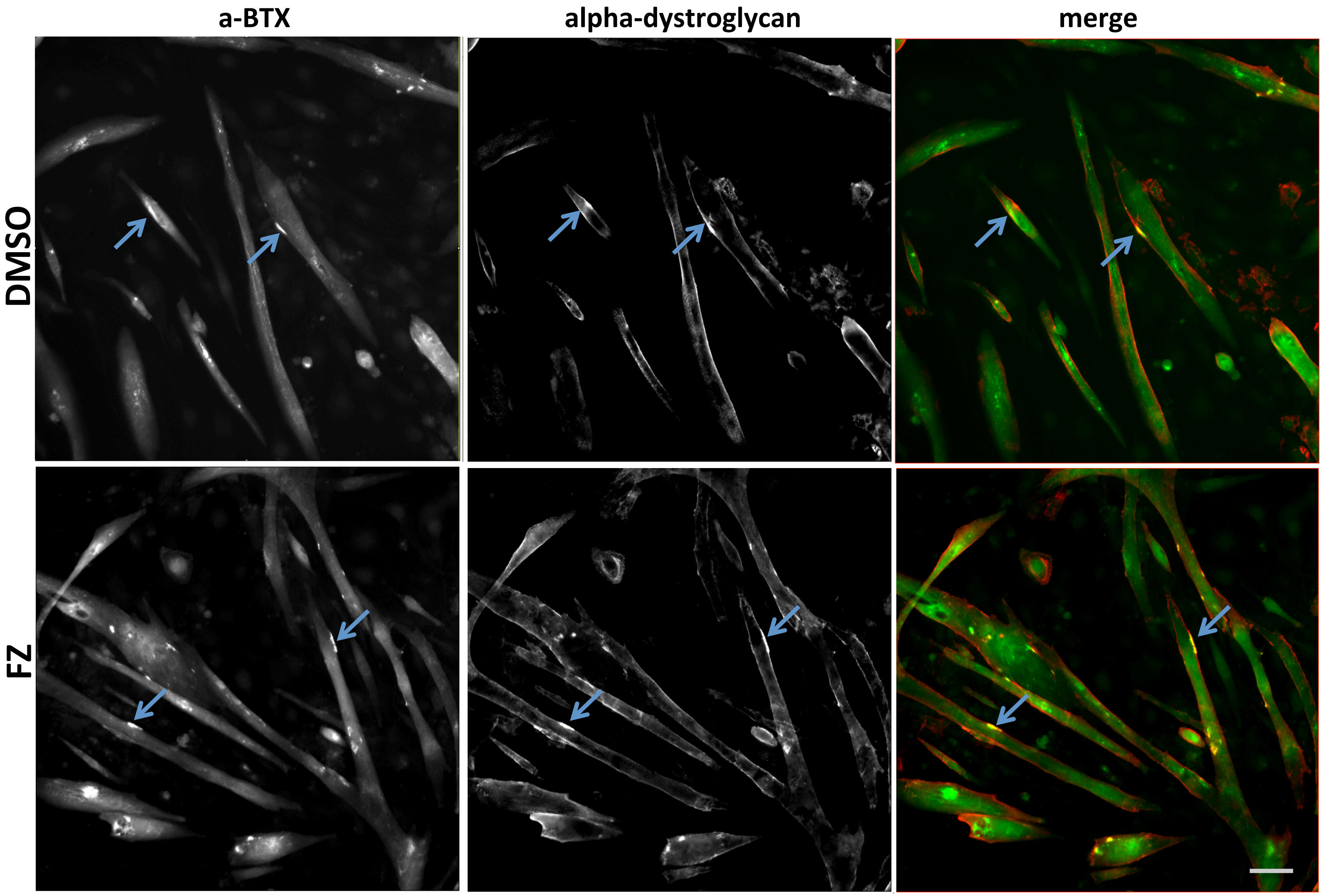

Supplemental Figure 4. Flunarizine accumulates dystroglycan with AChR clusters in C2C12 myotubes. Bar, 50 μm

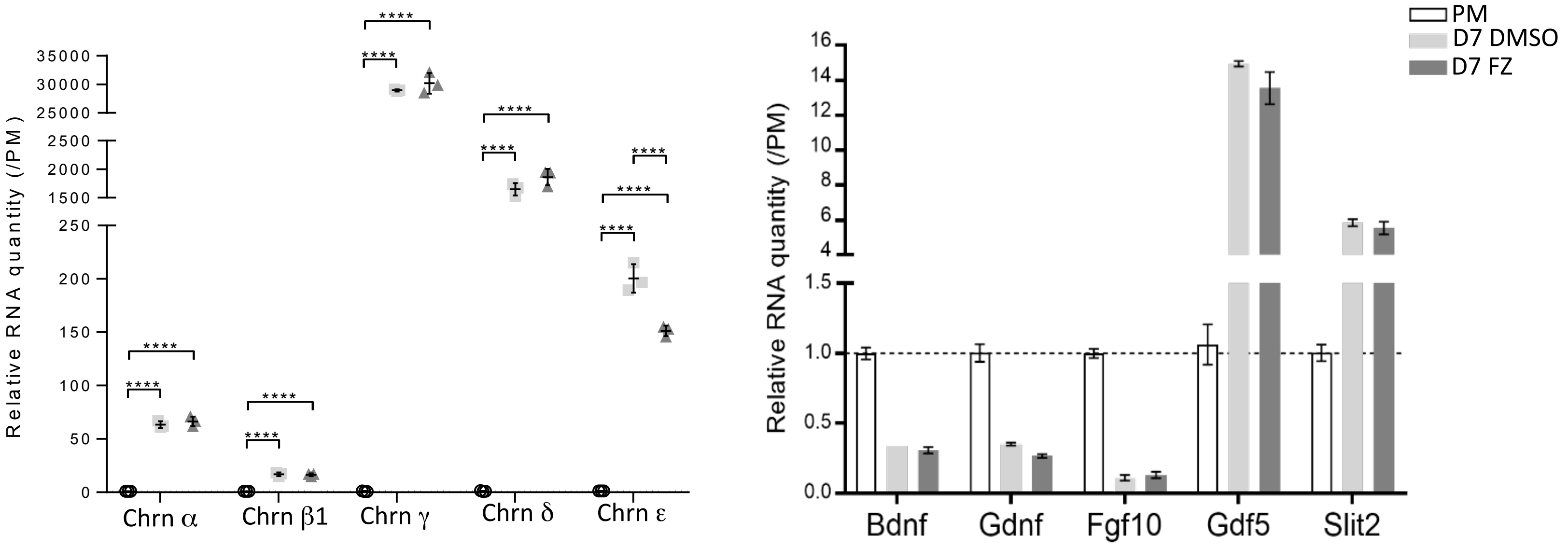

Supplemental Figure 5. Similar mRNA levels in DMSO and flunarizine-treated C2C212 myotubes. RT-qPCR analysis of AChR subunits and factors implicated in NMJ formation and YAP-dependent signaling (Zhao et al 2017 J Neurosci, 37: 3465-3477). The mRNA levels in C2C12 myotubes (Differentiated during 7 days, D7) are compared to levels expressed in C2C12 myoblast in proliferation medium (PM). PM is given an arbitrary value of 1.

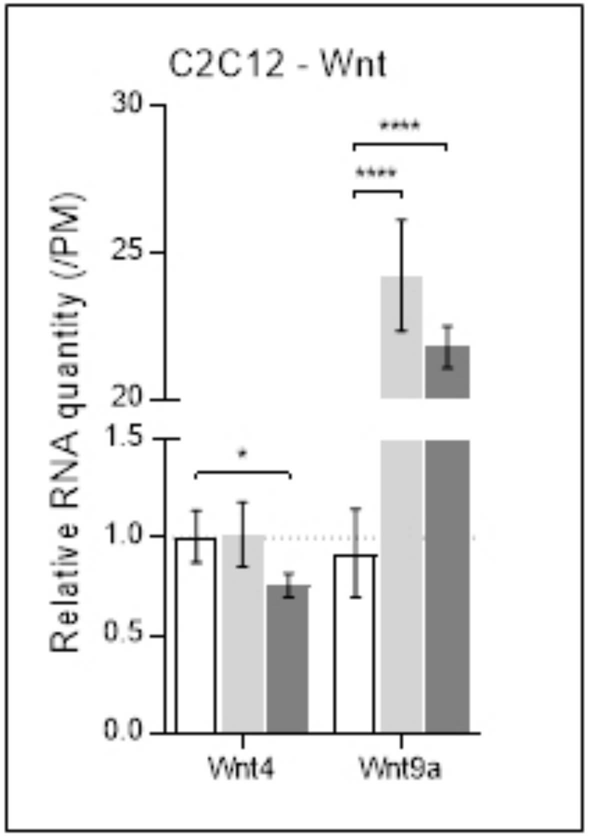

Supplemental Figure 6. Similar mRNA levels of Wnt4 and Wnt9a are expressed in DMSO and flunarizine-treated C2C212 myotubes . RT-qPCR analysis of Wnt molecules expressed in C2C12 myotubes and implicated in the NMJ formation (Shen et al 2018 elife 7:e34625). The mRNA levels in C2C12 myotubes (Differentiated during 7 days) are compared to levels expressed in C2C12 myoblasts cultured in proliferation medium (PM). PM is given an arbitrary value of 1. (n=3)

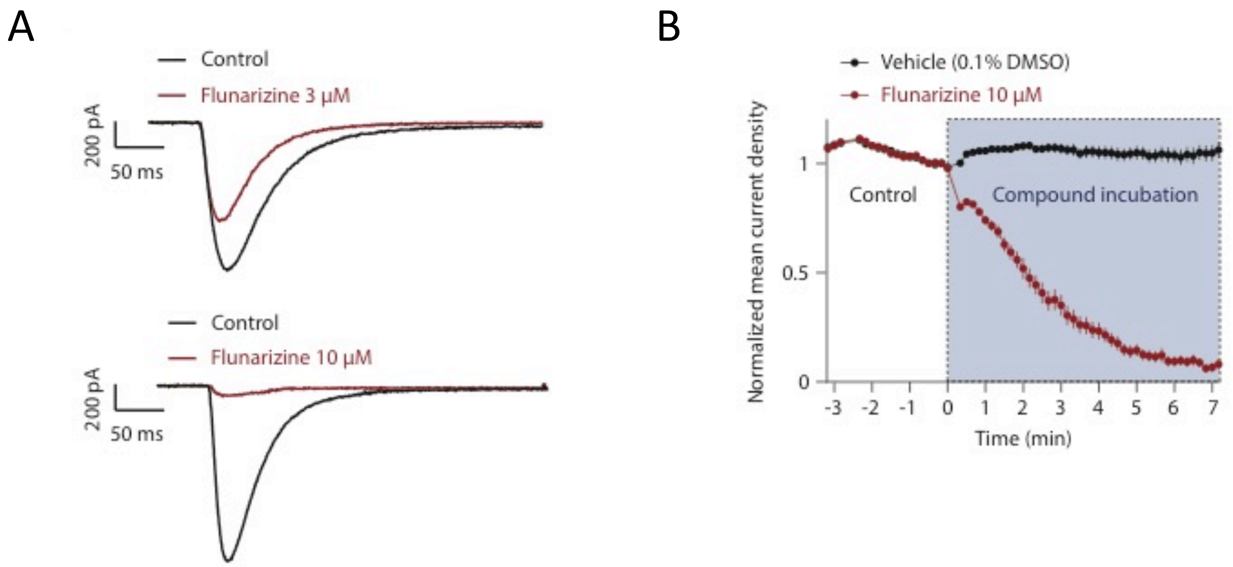

Supplemental figure 7. Flunarizine inhibits the Cav3.2 current. **A)** Representative traces showing Cav3.2 current blockade of a 3 µM (top) and a 10 µM (bottom) flunarizine application. **B)** 10 µM flunarizine inhibition time-course on Cav3.2 channels activity. Mean current densities were normalized to vehicle mean current densities.

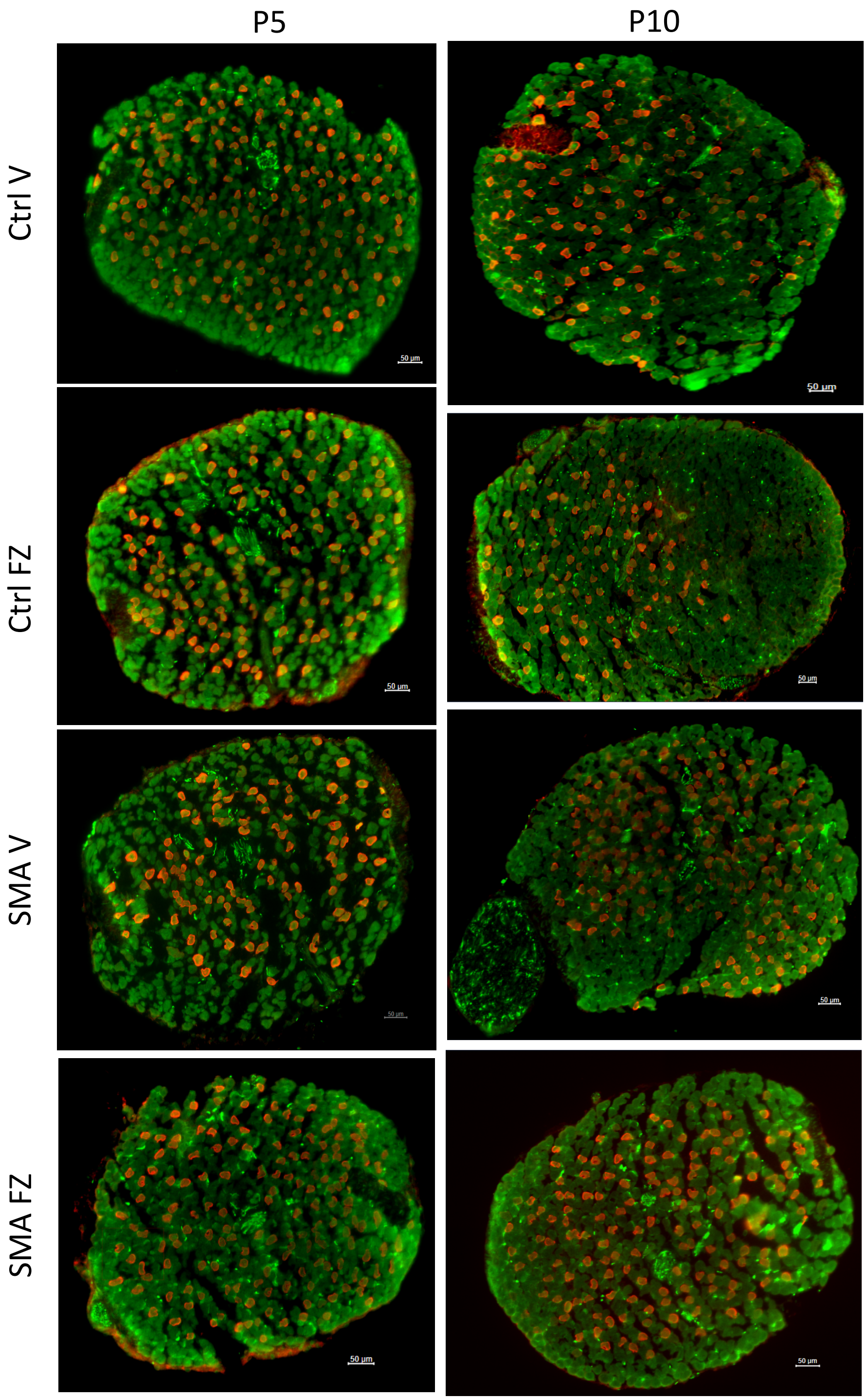

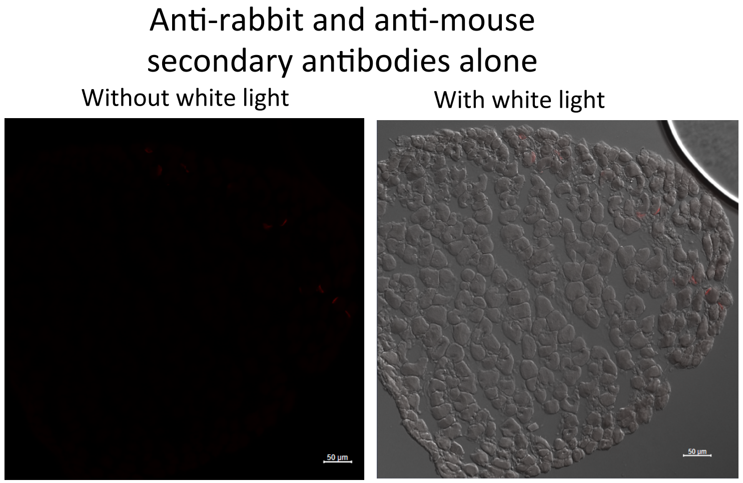

Supplemental Figure 8. Uncropped images of merge immunofluorescence results on sections of skeletal muscles presented in Figure 4 for the co-labelling of Cav3.2 (in green) and Type I fibers (in red). The secondary antibodies alone give no signal on muscle tissues. Scale bars, 50 µm.

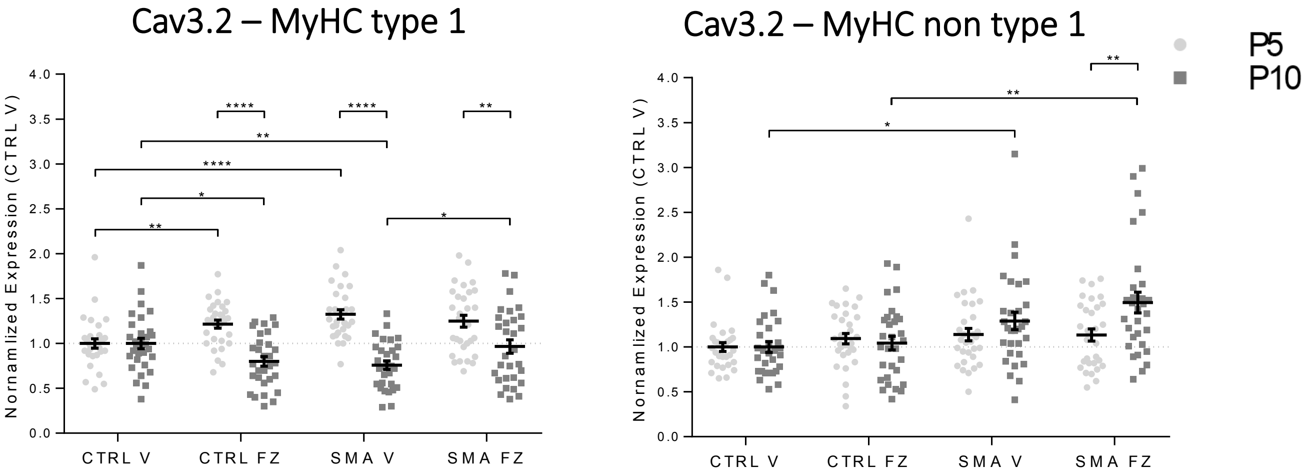

Supplemental Figure 9. Slow and fast neonatal fibers respond differently to flunarizine. Mean values of fluorescence intensity of individual muscle fibers for Cav3.2 detected in Figure 4A are shown. Each symbol represents the mean value of fluorescence intensity in a fiber. The distribution for the 4 groups of mice of MyHC7 immunolabelled or unlabeled fibers is compared using the student t-test. *P<0.05, **P<0.01, ***P<0.001 (3 mice per group).

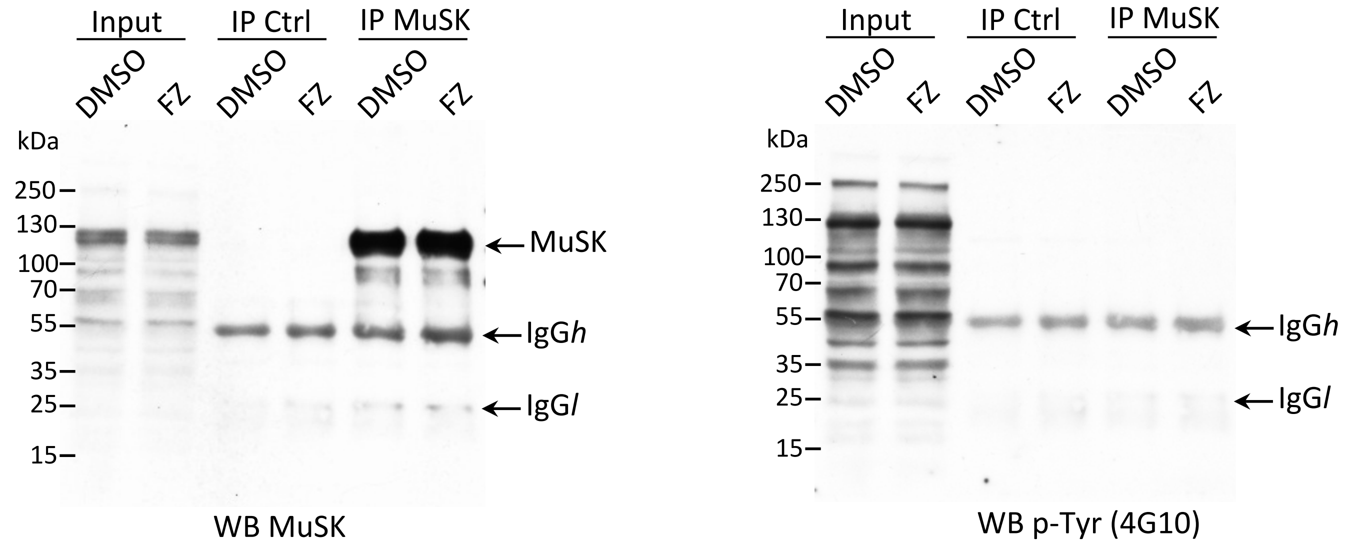

Supplemental figure 10. **Flunarizine effects is independent of an early MuSK tyrosine phophorylation.** Immunoprecipitations were performed with negative control mouse immunoglobulins (IP Ctrl) or anti-MuSK (IP MuSK) antibody with protein extracts from C2C12 myotubes treated 30 minutes with DMSO or flunarizine. Cells were lysed in RIPA buffer supplemented with protease and phosphatase inhibitors. Bound proteins were eluted from protein G-Dynabead, separated on SDS-PAGE (8%) with TRIS-Tricine running buffer and analysed by immunoblotting on PVDF membrane with anti-MuSK or anti-phosphotyrosine (p-Tyr) 4G10 antibody and Clean-Blot IP detection reagent. Bound proteins were compared to 5% input.

Supplemental table 1. Antibodies used for immunodetection studies

Target	Source	Host species	Concentration used		
			tissues	in vitro	immunoblot
SMN	#610646 (BD transduction laboratory)	mouse	-	-	1:1000
Unrip	ab46784 (Abcam)	mouse	-	-	1:400
AChR alpha	# 610988 (BD transduction laboratory)	mouse	-	-	1:5000
MuSK	ABS549 (Millipore)	rabbit	-	1:400	1:1000
LRP4	N207/27, #75-221 (NeuroMab)	mouse	-	1:400	1:1000
ITGB1	ab52971 (Abcam)	rabbit	-	-	1:1000
ITGB1	MAB1965 (Millipore)	mouse	-	1:400	-
ITGA11	ab198826 (Abcam)	rabbit	-	1:400	1:1000
Dyn2	ab65556 (Abcam)	rabbit	-	1:400	1:1000
CamKII total	Sc-13082 (Santa-Cruz)	rabbit	-	-	1:200
pCamKII	AB3865 (Chemicon)	rabbit	-	-	1:1000
GAPDH	MAB374 (Millipore)	mouse	-	-	1:10000
Tubulin alpha	T5168 (SigmaAldrich)	mouse	-	-	1:10000
$Ca_v3.2$	sc-25691 (H-300, Santa-Cruz) [specificity validated in Cav3.2-null mouse tissue by Garcia-Caballero et al 2014 Neuron 83, 1144-1158]	rabbit	1:400	-	1:1000
TXNIP	ab188865 (Abcam)	rabbit	1:400	-	1:2000
MyHC-I (*Myh7*)	M8421 (Sigma)	mouse	1:1000	-	-
alpha-Dystroglycan1	ab234587 (IIH6C4, Abcam)	mouse	-	1:100	1:200
beta-Dystroglycan1	Sc-33702 (Santa-Cruz)	mouse	-	-	1:200
phosphotyrosines	4G10 (Merck- millipore)	mouse		1:400	1:1000
Anti-mouse antibody (HRP)	GE Healthcare (NA931V)	sheep	-	-	1:25000
Anti-rabbit antibody (HRP)	GE Healthcare (NA934V)	donkey	-	-	1:30000
Anti-IgGk BP (HRP)	Sc-516102 (Santa-Cruz)	mouse	-	-	1:2000
Anti-rabbit antibody (alexa488)	A11034 (Invitrogen)	goat	1:400	-	-
Anti-rabbit antibody (Alexa488)	A11008 (Invitrogen)	goat	1:400	-	-
Anti-mouse antibody (Cy3)	#115-165-146 (Jackson Lab)	goat	1:400	-	-
Duolink PLA probe anti-mouse PLUS	DUO92001	-	-	1:5	-
Duolink PLA probe anti-rabbit MINUS	DUO92005	-	-	1:5	-
Anti-mouse IgM antibody (Alexa647)	A21238 (Invitrogen)	goat	1:200	-	-

Supplemental Table 2. Primers used for PCR and qPCR

Primer name	Sequence (5'-3')	Efficiency	R^2	Source
Agrin-NtA-F<br>Agrin-NtA-R	AGGAGATCCTCAACGTGGAC<br>TTACCACCTTGTTGCCACC	105	0.9934	herein
Agrin-Tm-F<br>Agrin-Tm-R	CCTGCAACATCTGCTTGATCC<br>TAGAAGGCGCTGCTGTGAAG	96	0.9996	herein
Agrin-ex5-6-F<br>Agrin-ex5-6-R	GCAAGAAGAATGTCTGCCC<br>AGTCAACACCATCACTGCC	81	0.9964	herein
Agrin-ex29-30-F<br>Agrin-ex29-30-R	GGGGGCTGCAATCATCAGG<br>GGCCCATCACCCACTTGAA	93	0.9987	primer bank ID20810012a1
Agrin-Y-F<br>Agrin-Y-R	GGCACCTGGGTTAGGGTATT<br>ACCTTGCGGGATTTCGGAG	98	0.9992	herein
Cacna1h-ex2-3-F<br>Cacna1h-ex2-3-R	TGCCACAGTCTTCTTCTGCCTC<br>AACGGCACTCAACATCCTCAC	85	0.9995	herein
Cacna1h-ex15-16-F<br>Cacna1h-ex15-16-R	CACCGATGAGGATAAGACATCTACC<br>ATTAGGGGTCACAGCCAGTGAG	90	0.9974	herein
Cacna1h-ex25-F<br>Cacna1h-ex25-R	GAGAACTTCCACAAGTGCC<br>AAGGTGATGAAGAGGTCCAG	84	0.9977	herein
Cacna1h-ex32-33-F<br>Cacna1h-ex32-33-R	TGGAGGAGAGCAACAAGGAG<br>ATTGGGTAGCGAGAGCATC	97	0.9985	herein
Txnip-F<br>Txnip-R	GTGTCCCTGGCTCCAAGAAA<br>GAGAGTCGTCCACATCGTCC	103	0.9769	herein
Wnt4-F<br>Wnt4-R	GAGAAGTGTGGCTGTGACCGG<br>ATGTTGTCCGAGCATCCTGACC	89	0.9988	Goodnough et al. 2014. Plos genet 10(2): e1004152. /doi.org/10.1371/journal.pgen.1004152
Wnt9a-F<br>Wnt9a-R	GCAGCAAGTTTGTCAAGGAGTTCC<br>GCAGGAGCCAGACACACCATG	101	0.9934	Goodnough et al. 2014. Plos genet 10(2): e1004152.
mBDNF-F1<br>mBDNF-R1	AGGCCAACTGAAGCAGTATTTCTAC<br>GAACATACGATTGGGTAGTTCGG	124	0.9892	Zhao et al. 2017. J Neurosci 37 (13) 3465-3477. doi.org/10.1523/JNEUROSCI.2934-16.2017
mFGF10-F1<br>mFGF10-R1	GCCACCAACTGCTCTTCTTCC<br>GCTGACCTTGCCGTTCTTCTC	108	0.9893	Zhao et al. 2017. Journal of Neuroscience 37 (13) 3465-3477.
mGDF5-F1<br>mGDF5-R1	GCCCACAAACCACGCAGTC<br>GCCACAAGATTCCACGACCAT	103	0.9608	Zhao et al. 2017. Journal of Neuroscience 37 (13) 3465-3477.
mGDNF-F1<br>mGDNF-R1	CTGACTTGGGTTTGGGCTATGA<br>TGCCTGGCCTACTTTGTCACTT	128	0.9962	Zhao et al. 2017. Journal of Neuroscience 37 (13) 3465-3477.
mSLIT2-F1<br>mSLIT2-R1	GGCAGACACTGTCCCTATCG<br>ATCTGTCTTCGTGATCCTCGTGA	108	0.9933	Zhao et al. 2017. Journal of Neuroscience 37 (13) 3465-3477.

Supplemental Table 3. Number of myotubes analysed for in-cell co-IP Duolink assays

Duo	LRP4-Cav3.2	LRP4-MuSK	Itgb1-MuSK	MuSK-pTyr	Itgb1-Itga11	Itgb1-Dyn2	total
Nber exp't	2	2	2	3	2	3	
dmso	120	148	186	351	141	322	1268
FZ	125	139	168	341	116	300	1189
							2457
